# A tetraoxane-based antimalarial drug candidate that overcomes PfK13-C580Y dependent artemisinin resistance

**DOI:** 10.1038/ncomms15159

**Published:** 2017-05-24

**Authors:** Paul M. O'Neill, Richard K. Amewu, Susan A. Charman, Sunil Sabbani, Nina F. Gnädig, Judith Straimer, David A. Fidock, Emma R. Shore, Natalie L. Roberts, Michael H.-L. Wong, W. David Hong, Chandrakala Pidathala, Chris Riley, Ben Murphy, Ghaith Aljayyoussi, Francisco Javier Gamo, Laura Sanz, Janneth Rodrigues, Carolina Gonzalez Cortes, Esperanza Herreros, Iñigo Angulo-Barturén, María Belén Jiménez-Díaz, Santiago Ferrer Bazaga, María Santos Martínez-Martínez, Brice Campo, Raman Sharma, Eileen Ryan, David M. Shackleford, Simon Campbell, Dennis A. Smith, Grennady Wirjanata, Rintis Noviyanti, Ric N. Price, Jutta Marfurt, Michael J. Palmer, Ian M. Copple, Amy E. Mercer, Andrea Ruecker, Michael J. Delves, Robert E. Sinden, Peter Siegl, Jill Davies, Rosemary Rochford, Clemens H. M. Kocken, Anne-Marie Zeeman, Gemma L. Nixon, Giancarlo A. Biagini, Stephen A. Ward

**Affiliations:** 1 Department of Chemistry, University of Liverpool, Liverpool L69 7ZD, UK; 2 Department of Pharmacology, School of Biomedical Sciences, MRC Centre for Drug Safety Science, University of Liverpool, Liverpool L69 3GE UK; 3 Centre for Drug Candidate Optimisation, Monash Institute of Pharmaceutical Sciences, Monash University, 381 Royal Parade, Parkville, Victoria 3052, Australia; 4 Department of Microbiology and Immunology, Columbia University College of Physicians and Surgeons, HHSC 1502, 701 W. 169th Street, New York, New York 10032, USA; 5 Division of Infectious Diseases, Department of Medicine, Columbia University Medical Center, HHSC 1502, 701 W. 168th Street, New York, New York 10032, USA; 6 Research Centre for Drugs and Diagnostics, Liverpool School of Tropical Medicine, Pembroke Place, Liverpool L3 5QA, UK; 7 Tres Cantos Medicines Development Campus, DDW, GlaxoSmithKline, Severo Ochoa 2, 28760 Tres Cantos, Spain; 8 Medicines for Malaria Venture, ICC, Route de Pré-Bois 20, P.O. Box 1826, 1215 Geneva, Switzerland; 9 Global and Tropical Health Division, Menzies School of Health Research, Charles Darwin University, P.O. Box 41096, Casuarina, Darwin, Northern Territory 0811, Australia; 10 Eijkman Institute for Molecular Biology, Jl. Diponegoro 69, 10430 Jakarta, Indonesia; 11 Nuffield Department of Clinical Medicine, Centre for Tropical Medicine and Global Health, University of Oxford, Old Road Campus, Roosevelt Drive, Oxford OX3 7FZ, UK; 12 Department of Life Sciences, Imperial College London, South Kensington, London SW7 2AZ, UK; 13 Nuffield Department of Medicine, The Jenner Institute, University of Oxford, Old Road Campus Research Building, Roosevelt Drive, Oxford, OX3 7DQ, UK; 14 Department of Immunology and Microbiology, University of Colorado, Aurora Colorado, CO 80045, USA; 15 Department of Parasitology, Biomedical Primate Research Centre, P. O. Box 3306, 2280 GH Rijswijk, The Netherlands

## Abstract

K13 gene mutations are a primary marker of artemisinin resistance in *Plasmodium falciparum* malaria that threatens the long-term clinical utility of artemisinin-based combination therapies, the cornerstone of modern day malaria treatment. Here we describe a multinational drug discovery programme that has delivered a synthetic tetraoxane-based molecule, E209, which meets key requirements of the Medicines for Malaria Venture drug candidate profiles. E209 has potent nanomolar inhibitory activity against multiple strains of *P. falciparum* and *P. vivax in vitro*, is efficacious against *P. falciparum* in *in vivo* rodent models, produces parasite reduction ratios equivalent to dihydroartemisinin and has pharmacokinetic and pharmacodynamic characteristics compatible with a single-dose cure. *In vitro* studies with transgenic parasites expressing variant forms of K13 show no cross-resistance with the C580Y mutation, the primary variant observed in Southeast Asia. E209 is a superior next generation endoperoxide with combined pharmacokinetic and pharmacodynamic features that overcome the liabilities of artemisinin derivatives.

## Abstract (web summary)

Artemisinin-resistant *Plasmodium* is an increasing problem. Here, using a medicinal chemistry programme, the authors identify a tetraoxane-based drug candidate that shows no cross-resistance with an artemisinin-resistant strain (PfK13-C580Y) and is efficient in *Plasmodium* mouse models.

The control and elimination of malaria requires effective treatment strategies and the universal availability of artemisinin-based combination therapies (ACTs)^1^,^2^. For over a decade, research groups have sought to replace the semi-synthetic artemisinin components (Fig. 1a, **1a–1c**) of ACTs with a fully synthetic alternative to reduce uncertainties regarding cost and supply and to improve overall pharmacological qualities, most notably short clinical half-lives. Several pharmacophores have been explored^3^ including the tetraoxanes (Fig. 1b, **3a** and **3b**).

Recent years, however, have witnessed the emergence of parasite ‘resistance' to artemisinin, first documented in western Cambodia^4^,^5^. Malaria patients from this region were reported to have a delayed parasite clearance following either artesunate monotherapy or an ACT. The delayed parasite clearance phenotype is not resistance as defined by the WHO^6^,^7^ and does not necessarily lead to treatment failure^8^. However, it gives rise to concerns of a reduction in the therapeutic lifespan of the most effective, currently registered anti-malarials and the only class that offers rapid parasite biomass reduction. Furthermore, reduced artemisinin efficacy places greater selective pressure on the ACT partner drugs, increasing the risk of multidrug resistance to emerge. Indeed, recent results in Cambodia now document increasing rates of clinical treatment failure in patients treated with the first-line combination dihydroartemisinin plus piperaquine, because of the emergence of resistance to both agents^9^

Here we describe the development of a fully synthetic tetraoxane analogue, E209, which displays nanomolar efficacy against multiple strains of *P. falciparum* and *P. vivax* with a tailored combination of pharmacodynamic (PD) and pharmacokinetic (PK) properties compatible with a single-dose cure even against a backdrop of the K13-dependent resistance mechanism in operation in S.E. Asia^8^.

## Results

### Optimization of the tetraoxane series

**Figure 1:**
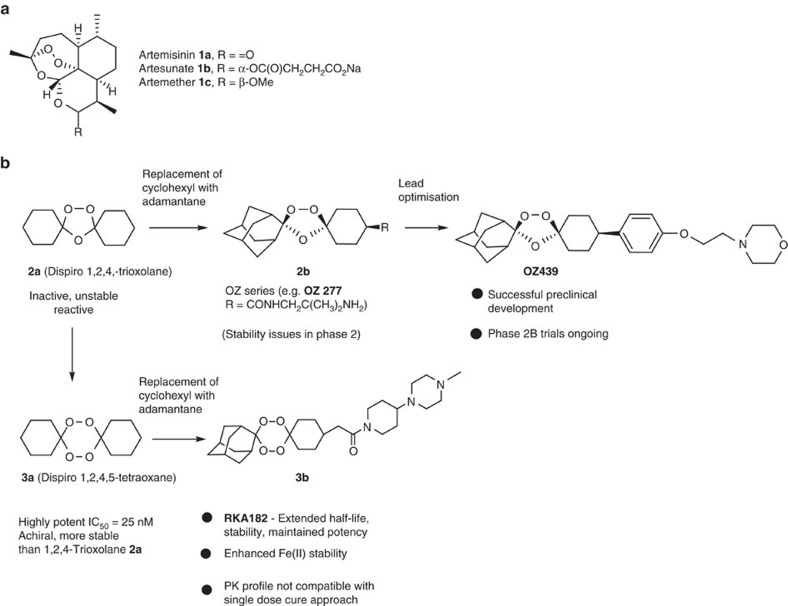
Artemisinins and synthetic peroxides. (**a**) Artemisinin and semi-synthetic analogues. (**b**) Comparison of tetraoxanes with trioxolane-based anti-malarials. MSD in days following a single oral dose of 30 mg kg^−1^, Pf SCID (NOD-SCID IL-2Rγc^*null*^ (NSG) mice engrafted with human erythrocytes and infected with *P. falciparum* strain 3D7^0087/N9^).

The tetraoxane core **3a** is differentiated from 1,2,4-trioxolanes **2a** and 1,2,4-trioxanes as it is achiral, has greater inherent thermodynamic stability and potent *in vitro* antimalarial activity, either as a simple unsubstituted analogue, for example, **3a** (Fig. 1) or related derivatives. Recent theoretical calculations based on stereoelectronic analysis suggest that the remarkable thermodynamic stability of tetraoxanes can be attributed to a stereoelectronic ‘double anomeric effect' which stabilizes the six-membered ring system^10^.

Our initial medicinal chemistry focus was the preparation of variants of a simplified analogue of our previous candidate RKA 182 with more balanced ADME properties (**3b**)^11^ (Supplementary Figs 1, 2, 3 and 4). Multiple series were prepared to modulate solubility, metabolic stability, pKa and log D (Supplementary Fig. 4) but in all cases, the amide linked analogues, although superior to the artemisinins, had relatively short half-lives and poor antimalarial activity in the *Plasmodium berghei* mouse model. We next prepared tetraoxane **4** as a direct comparator (Supplementary Fig. 4; Supplementary Table 1) to OZ439 (ref. 12), which is currently in phase 2 clinical trials. Although this analogue had equivalent *in vitro* potency to OZ439, it only achieved a 16.3 day mean survival time (MSD) at a single 1 × 30 mg kg^−1^ oral dose in *P. berghei* infected mice (Table 1). This molecule was also slightly less stable in microsomal studies *in vitro* with high clearance rates and a short half-life in rat PK studies.

Cognisant of the liabilities of the artemisinin class, single-dose survival time, intrinsic clearance and blood stability were used as the key parameters to drive SAR studies towards lead series progression (Supplementary Fig. 4; Supplementary Information). Representative lead compounds are shown in Supplementary Fig. 4 from which E209 and N205 were the clear front-runner molecules with respect to cures in the *P. berghei* model and initial DMPK assessments (Supplementary Table 1; Supplementary Fig. 5) and were selected for further profiling. *In vitro* metabolic stability assessments (Supplementary Table 2) and solubility assessments (Supplementary Table 3) demonstrated the superior properties of E209 and this compound was taken forward.

Following scale up synthesis (Supplementary Note 2; Supplementary Fig. 1), E209 was progressed to candidate selection through a series of chemical, parasitological, pharmacological and toxicological checkpoints as outlined below.

### PD properties of E209

E209 retained potent *in vitro* efficacy (mean IC_50_ range 2.9–14.0 nM) against a panel of 10 sensitive and multidrug-resistant *P. falciparum* parasite isolates from distinct geographical origins, with no observable cross-resistance in growth inhibition studies (Supplementary Table 4). Supplementary Table 4 represents a panel of parasite isolates used as part of a standard Medicines for Malaria Venture (MMV) *in vitro* evaluation and includes parasites that are universally sensitive to current drugs (HB3) and parasites resistant to chloroquine (as indicated), mefloquine (FCB, NF54), pyrimethamine and related DHFR inhibitors (K1, TMC90, V1/S and so on) and atovaquone (TMC90). In the case of E209, activity against all parasites is below the accepted cut off for resistance and it is clear from visual inspection that there is no relationship at all with chloroquine sensitivity. Cytotoxicity studies using HeLa cells demonstrated that E209 had a high *in vitro* therapeutic index of 200–1,000, with a median IC_50_ 2.85 (s.d.±0.99 μM, *n*=3) (*c.f.* dihydroartemisinin cytotoxicity (median IC_50_ 0.4 (s.d.±0.1 μM, *n*=3)).

*Ex vivo P. falciparum* and *P. vivax* drug sensitivity experiments were carried out in Papua Province in eastern Indonesia, an area with documented multidrug-resistant *P. falciparum* and CQ-resistant *P. vivax*, using a modified schizont maturation assay as described previously^13^,^14^,^15^,^16^,^17^. Ethical approval for the *ex vivo P. falciparum* and *P. vivax* efficacy data was obtained from the Human Research Ethics Committee of the NT Department of Health & Families and Menzies School of Health Research, Darwin, Australia (HREC 2010-1396) and the Eijkman Institute Research Ethics Commission, Jakarta, Indonesia (EIREC 47 and EIREC 67). E209 exhibited equipotent *ex vivo* activity against *P. vivax* (*n*=13; median IC_50_: 10.5 nM) and *P. falciparum* (*n*=3; median IC_50_: 15.7 nM) clinical field isolates from Papua, Indonesia (Mann–Whitney *U* test, *P*=0.570); Supplementary Table 5. With the exception of chloroquine in *P. falciparum* (Spearman rank correlation, *P*=0.042), there was no correlation between the activity of E209 and any of the other anti-malarials assessed in either species ((amodiaquine (AQ), piperaquine (PIP), mefloquine (MFQ) and artesunate (AS) (Supplementary Table 6)).

E209 was screened for late stage gametocytocidal activity in a standard membrane feeding assay (SMFA) and transmission-blocking activity was determined in *Anopheles stephensi* mosquitoes. Late stage gametocytes were incubated with E209 for 24 h prior to mosquito feeding and the IC_50_, (determined by the reduction in oocyst mean intensity) was 14.5 nM with a corresponding ∼60 and 90% transmission-blocking activity (defined as the percentage of mosquitoes with no observed oocysts) recorded at 100 nM and 1 μM, respectively (Supplementary Fig. 6). The transmission reducing activity (TRA) shown by E209 is consistent with that shown for the endoperoxide class as reported in recent SMFA-based studies^18^,^19^.

**Figure 2:**
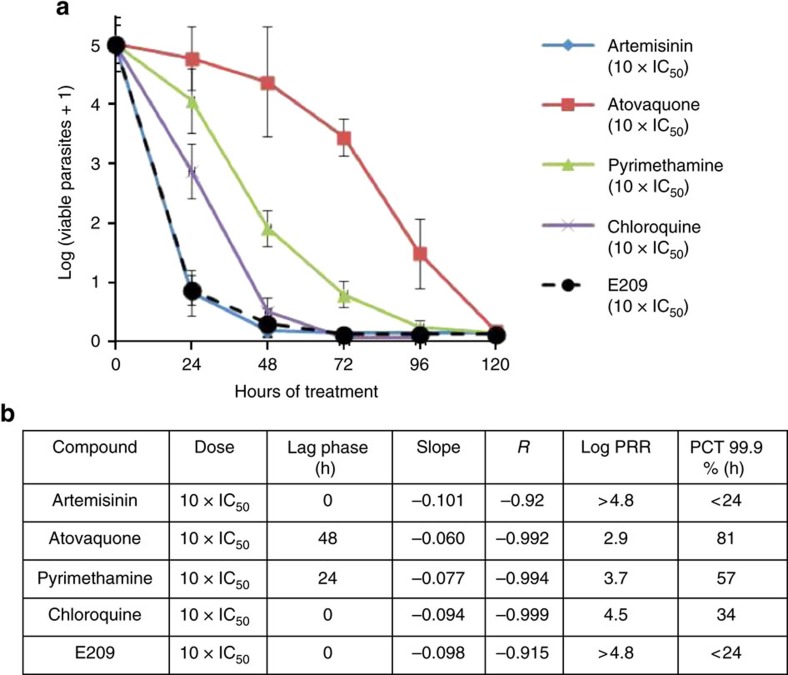
Parasite reduction ratios of clinically used anti-malarials and E209. (**a**) *In vitro* PRR—number of viable parasites after E209 treatment (10 × IC_50_) is compared with the profile shown by standard anti-malarials (**b**) Lag phase, log_10_PRR and parasite clearance time (PCT) values for E209 and a selection of standard anti-malarials. The *in vitro* parasite reduction rate assay was used to determine onset of action and rate of killing as previously described^20^. *P. falciparum* was exposed to E209 at a concentration corresponding to 10 × EC_50_. The number of viable parasites at each time point was determined as described by Sanz *et al*.^20^ Four independent serial dilutions were done with each sample to correct for experimental variation; error bars, s.d. Previous results reported on standard anti-malarials tested at 10 × EC_50_ using the same conditions are shown for comparison.

E209 (10 × IC_50_) displayed fast killing kinetics (Supplementary Methods), with an *in vitro* parasite reduction ratio (log_10_PRR) >4.8 similar to artemisinin (log_10_PRR>4.8), and faster than CQ (log_10_PRR 4.5), pyrimethamine (log_10_PRR 3.7) and atovaquone (log_10_PRR 2.9) (Fig. 2)^20^.

**Figure 3:**
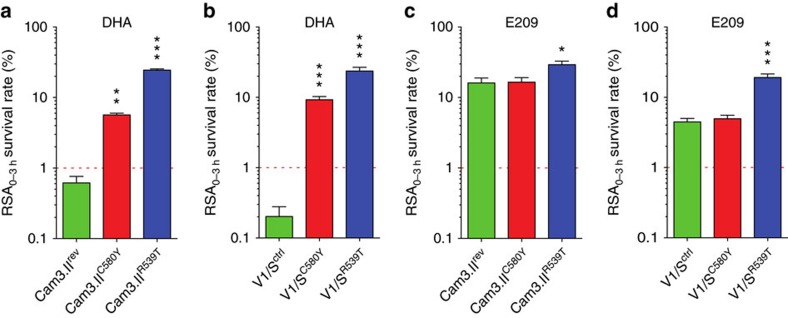
Ring-stage survival assays show equal potency of E209 against K13 wild-type and C580Y mutant parasites whereas R539T confers a marginal increase of survival. Results show the percentage of early ring-stage parasites (0–3 h post-invasion of human erythrocytes) that survived a 6-h pulse of 700 nM DHA or E209, as measured by flow cytometry 90 hours later. Data show mean±s.e.m. percentage survival compared to DMSO-treated parasites processed in parallel. Assays were performed on at least 2 separate occasions in duplicate. K13-propeller mutations R539T and C580Y confer resistance to DHA in the clinical isolate Cam3.II and the reference line V1/S (**a**), (**b**). Exposure to E209 reveals generally higher survival with slight cross-resistance observed in parasites carrying the R539T mutation (**c**), (**d**). A two-sample t-test with unequal variances was used to calculate *P* values. These can be found in Supplementary Tables 7–14. *P value<0.05, **P value<0.001, ***P value<0.0001.

Critical for the development of E209 and related structures is the evaluation of their activity against ART-resistant parasites, which has been defined as delayed parasite clearance in patients^5^.This phenotype has been associated clinically with a number of non-synonymous SNPs in the propeller domain of the K13 protein, located on chromosome 13 (ref. 23). Notably, these mutations could not be associated with ART resistance using the standard *in vitro* 72 h inhibitory assay, therefore a novel phenotypic assay was developed to assess ART resistance *in vitro.* The ring-stage survival assay (RSA_0–3 h_) is based on the increased resistance of young ring-stage parasites to a 6 h pulse of 700 nM dihydroartemisinin (DHA)^21^,^22^. To investigate the impact of *K13* mutations on E209 susceptibility, a series of gene-edited, otherwise isogenic parasite lines with either K13 mutant or wild-type alleles have been subjected to RSA_0–3 h_ assays using DHA as comparison^23^. This allowed us to assess the potency of a pharmacological relevant dose of E209 against K13 mutant parasites and to identify potential cross-resistance phenotypes. In this study we focused on the most prevalent mutation C580Y and R539T, which was demonstrated to confer high levels of DHA resistance *in vitro*^23^(Fig. 3).Generally, we observe higher survival rates after exposure of young ring stages to E209 in both parasite backgrounds when compared to DHA. When subjected to a 700 nM dose for 6 h, we observe 4% survival of V1/S and 16% of Cam3.II parasites for E209 compared to <1% survival when pulsed with DHA. The highly prevalent *K13* mutation C580Y does not confer cross-resistance to E209. However, R539T shows a moderate but significant increase of parasite survival in both parasites lines tested. (Ring-stage survival assays, fold changes between K13 wild-type and K13-mutant lines and IC_50_ data for DHA and E209 are recorded in Supplementary Tables 15 and 16; Supplementary Fig. 7).

**Figure 4:**
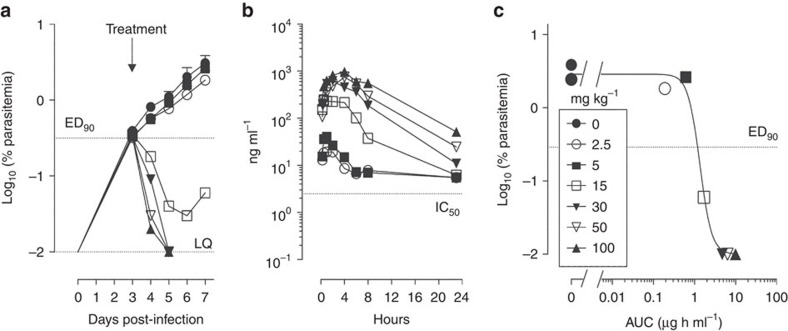
Efficacy of E209 in the *in vivo*-humanized SCID mouse model of *P. falciparum*. (**a**) Parasitemia in peripheral blood of NSG mice infected with the *P. falciparum* strain 3D7^0087/N9^ (*n*=2 mice treated with vehicle and *n*=6 mice treated with E209). (**b**) Blood levels of E209 in the efficacy experiment shown in **a** over the first 24 h post-dosing. (**c**) dose/exposure–response relationship.

*In vivo* experiments using *P. berghei* infected mice demonstrated that E209 when given orally resulted in complete parasite clearance in the Peters' standard 4-day suppressive test^24^, with an estimated ED_50_ of 4 mg kg^−1^ after three doses. Importantly, E209 also achieves 66% cure rate after a single oral dose of 30 mg kg^−1^ (Table 1). Efficacy of E209 against the human malaria parasite was tested in the immune-deficient NOD-SCID IL-2R_null mouse engrafted with human erythrocytes and infected with the *P. falciparum* strain 3D7^0087/N9^ (ref. 25). Complete parasite clearance was achievable with a single oral dose of E209 (30 mg kg^−1^) with a calculated ED_90_ of 11.6 mg kg^−1^ and an AUC ED_90_ of 1.2 μg h ml^−1^ (Fig. 4). This compares with an ED_90_ of 10 mg kg^−1^ for artesunate following four consecutive daily doses^26^. The maximum rate of parasite killing by E209 was achieved with blood exposure between 5 and 10 μg h ml^−1^ (Fig. 4). Therefore, this range of exposure in human blood is predicted to be the lowest necessary to achieve the maximum parasite killing and can therefore be defined as the minimum parasiticidal concentration (MPC).

### *In vitro* and *in vivo* PKs of E209

E209 exhibited metabolic degradation in human, rat and mouse liver microsomes with rates generally being fastest in rat and slowest in the mouse (Supplementary Table 17; Table 2). Comparing metabolite profiles across all three species, hydroxylation(s) on the adamantane ring to form multiple M+16 metabolites represent important pathways in all three species. In addition, *N*-oxidation to M+16 appears to be an important metabolic pathway in rat whilst tetraoxane cleavage products were observed in all three species (Supplementary Tables 17, 18, 19; Supplementary Fig. 8).

**Figure 5:**
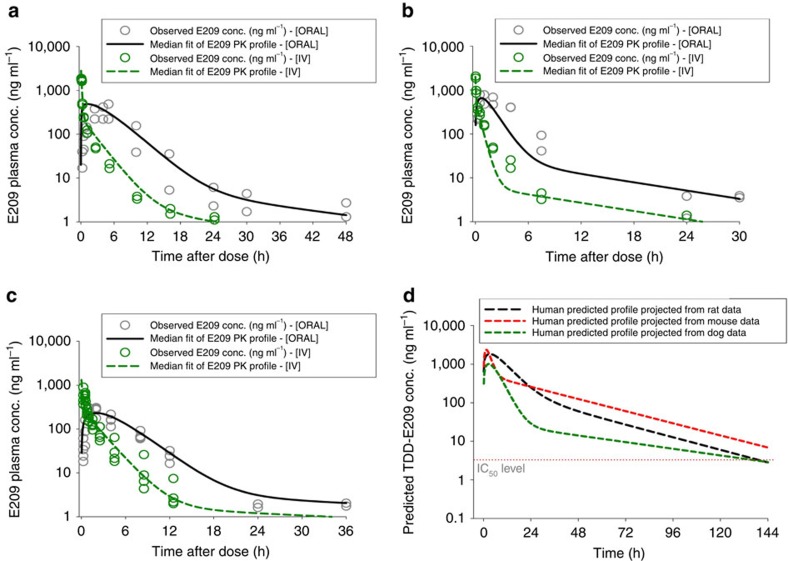
Measured rodent and predicted human exposure profiles. PK profiles of E209 in male Sprague–Dawley rats (**a** oral dose=9 mg kg^−1^, IV dose=2 mg kg^−1^), outbred female Swiss mice (**b** oral dose=10 mg kg^−1^, IV dose=2 mg kg^−1^) and the male beagle dog (**c** oral dose=5 mg kg^−1^, IV dose=1 mg kg^−1^) and allometrically scaled predictions for human PK (**d**) based on all of the above assuming an oral dose of 15 mg kg^−1^. Fits constructed are based on predicted median PK parameter values.

Compartmental PK modelling of preclinical *in vivo* data showed a clear two compartmental trend for all three species. Simulated PK profiles based on these compartmental model predictions (Table 2) showed good agreement with observed data as shown in Fig. 5a–c). Allometric scaling (see Methods) rat PK data predicted a terminal half-life in humans of 24–30 h and drug levels that exceed E209 IC_50_ levels for up to 6 days following a single oral dose of 15 mg kg^−1^ (Fig. 5d).

### Preclinical safety studies

To assess potential for adverse activities, we profiled E209 using a diverse panel of radioligand binding assays (CEREP screen), ion channel voltage clamp assays and genotoxicity assays. In the CEREP screen, E209 at 10 μM demonstrated a favourable profile apart from moderate potency at Sigma 1 and Sigma 2 receptors (IC_50_s *ca*. 200 nM). Risk of adverse cardiac activity including QT interval prolongation is predicted to be low based on the measurement of a high IC_50_ (>3.5 μM) in the hERG assay and no inhibitory activity at >100 μM in Cav1.2 and Nav1.5 assays. E209 was negative in the Ames test and mouse micronucleus test confirming the absence of mutagenic potential.

We also examined the potential haemolytic effect of E209 in NOD-SCID mice engrafted with the African variant G6PD deficient human red blood cells (huRBC) as previously reported for primaquine^27^. Mice treated with E209 at doses up to 50 mg kg^−1 ^day^−1^ for 3 days did not show a significant drug-dependent loss of huRBC at 7 days post-treatment with all test groups demonstrating a response similar to the vehicle control (Supplementary Fig. 9A–C). In contrast primaquine, a drug that causes haemolytic toxicity in humans with G6PD deficiency, induced significant loss of huRBC (Supplementary Fig. 9A–C).

In addition, *In vitro* studies with cytochrome P450 (CYP) isoforms demonstrated that E209 did not inhibit any of the major human CYP450s with IC_50_s >20 μM (Supplementary Table 20).

The maximum-tolerated dose (MTD) following a single oral administration of E209 in Sprague–Dawley rats was 300 mg kg^−1^. A 7-day repeat dose, exploratory toxicity study in rats was performed using dose levels of 50,100 and 200 mg kg^−1^ and parallel groups for toxicokinetic analysis. At 200 mg kg^−1 ^day^−1^ E209 was not tolerated in either sex. At 100 mg kg^−1^ there was body weight loss in females. On the basis of weight loss and histopathology findings, the no observed adverse effect level (NOAEL) for this study was 100 mg kg^−1 ^day^−1^ for males and 50 mg kg^−1 ^day^−1^ for females. Systemic exposure values (*C*_max_ and AUC_0–24 h_) at the NOAEL on day 7 were 1.2 μg ml^−1^ and 19.6 μg h ml^−1^, respectively, in males and 1.07 μg ml^−1^ and 14.8 μg h ml^−1^, respectively, in females. Additional single and multiple dose safety pharmacology studies are in progress in rodent and non-rodent models to establish toxicity profile and predicted exposure multiple at NOAEL over target therapeutic exposure (therapeutic index).

## Discussion

A systematic evaluation of the antimalarial tetraoxane scaffold (Supplementary Note 1), revealed the aryloxy template (Fig. 1) to be the most promising in terms of multi-parameter lead-optimization. Through a series of rational chemical modifications we have been able to improve both the inherent pharmacodymamics (*in vitro* and *in vivo* potency) and PKs (metabolic stability profiles in a range of species and rodent and human blood stability) of the class.

In addition to overall high potency, our studies also demonstrate that the fast PRR for E209 observed in *in vitro* studies translates to a fast action *in vivo* in the humanized SCID mouse model where a maximum parasite clearance was achieved after a single dose of 30 mg kg^−1^. To achieve this level of parasite clearance with the clinical gold standard endoperoxide artesunate requires four doses of artesunate (50 mg kg^−1^ day^−1^) in the same model^25^. Initial safety and pharmacology profiles for E209 meet the requirements of MMV's target candidate profile 1 (TCP1), a drug with a fast killing profile (PRR equivalent to or better than dihydroartemisinin) and PK qualities that would support a single-dose cure either singly or in combination.

It is generally accepted that the mechanism of action of endoperoxide anti-malarials involves intra-parasitic ferrous mediated reductive scission of the endoperoxide bond. This process leads to the generation of reactive oxygen species such as carbon-centered radicals, which react with multiple parasite proteins and result in parasite killing^28^,^29^,^30^. Because of this proposed mechanism of action it was originally argued that malaria parasites would find it difficult to acquire resistance to this drug class. However, compelling clinical data from Southeast Asia has confirmed that resistance has emerged and established itself in the region. The resistance phenotype has been difficult to study, as it does not show up as a shift in potency (IC_50_ value) in traditional drug sensitivity assays^20^. Extensive molecular investigations have implicated mutations in the *K13* gene as key to this unusual resistance phenotype. These mutations allow the parasite to survive exposure to drug at the early ring stage of red blood cell infection even at supra-pharmacological drug concentrations^20^,^21^. A concern is that this type of resistance mechanism would blight the long-term clinical utility of the entire endoperoxide class of antimalarial drugs. Our interpretation of this resistance mechanism, which is supported by recent modelling studies^28^, is that the slow parasite clearance phenotype seen clinically is a result of the loss of drug susceptibility in ring-stage parasites coupled with the extremely short elimination half-life of the endoperoxide. A potential solution to this problem is to extend the current 3-day artemisinin treatment course to four days as suggested by Dogovski *et al*.^31^ We believe that E209 provides a more elegant solution since it circumvents or minimizes the ring-stage resistance seen with currently deployed endoperoxides with a predicted elimination half-life of 24–30 h compared to current artemisinins that have half-lives of circa 1–2 h (ref. 32). Hence, not only will E209 plasma levels remain above the IC_50_ level for 4 days or more in malaria patients as required by MMV's TCP1 criteria (based on PK predictions, Supplementary Methods), but it will also retain killing potential throughout each individual stage of parasite's 48 h intra-erythrocytic cycle. Thus, E209 has the potential for deployment in a superior combination treatment with a partner drug devoid of existing *in vivo* resistance liabilities (such as the ATP4 inhibitors^33^,^34^ or DHODH inhibitors^35^). E209 has the potential to offer a substantial improvement on currently deployed artemisinin-based drug combinations and provide an urgently required alternative TCP1 drug for malaria treatment and elimination programmes.

## Methods

### Synthesis of E209

Details of the synthesis and analytical data on E209 are included in Supplementary Note 2.

### Parasite culture and drug sensitivity testing

*P. falciparum* blood stage cultures were maintained by the method of Trager and Jensen^36^. Drug sensitivity during E209 development and QSAR was determined by the method of Winter *et al*.^37^ Drug sensitivity of E209 against panel of *P. falciparum* strains was determined based on hypoxanthine incorporation^38^,^39^. Mammalian cell cytotoxicity assays were carried out as previously described^40^.

A zinc-finger nuclease (ZFN)-based gene editing approach was employed to specifically introduce or remove K13 mutations from *P. falciparum* lines. Parasites were electroporated with a plasmid containing ZFNs specifically engineered against *K13*. The nucleases were expressed from an episomally maintained plasmid by selecting with 2.5 nM (Cam3.II) or 10 nM (V1/S) WR99210 for 6 days. A 1.5 kb K13 donor fragment harbouring the desired mutation was placed on the same plasmid and served as template for the double strand break repair. Parasite clones were generated by limiting dilution. A detailed protocol can be found in Straimer *et al*.^21^

Ring-stage survival assays (RSA_0–3 h_) were carried out as previously described with minor modifications^21^,^23^. In summary, 10–15 ml parasite cultures were synchronized 1–2 times using 5% sorbitol (Sigma-Aldrich). Synchronous multinucleated schizonts were incubated in RPMI-1640 containing 15 units/ml sodium heparin for 15 min at 37 °C to disrupt agglutinated erythrocytes (purchased from Interstate Blood Bank located in Memphis, TN, USA), concentrated over a gradient of 75% Percoll (Sigma-Aldrich), washed once in RPMI-1640 and incubated for 3 h with fresh erythrocytes to allow time for merozoite invasion. Cultures were then subjected again to sorbitol treatment to eliminate remaining schizonts. The 0–3 h post-invasion rings were adjusted to 1% parasitemia and 2% haematocrit in 1 ml volumes (in 48-well plates), and exposed to 700 nM DHA, TDD-E209, JC3-39 or 0.1% DMSO (solvent control) for 6 h. Duplicate wells were established for each parasite line±drug. The supernatant was then removed, the culture resuspended in 1 ml culture media and transferred to 15 ml conical tubes, centrifuged at 800*g* for 3 min to pellet the cells and the supernatants carefully removed. As a washing step to remove drug, 10 ml culture medium were added to each tube and the cells resuspended, centrifuged and the medium aspirated. This washing procedure was repeated three times and included a transfer into a new 15 ml conical tube after the second washing step. Fresh medium lacking drug was then added to cultures, which were returned to standard culture conditions for 90 h. Parasite viability was assessed by microscopic examination of Giemsa-stained thin blood smears. Second-generation ring stages are clearly visible under the microscope but only give a faint signal when measured by flow cytometry. Therefore, we performed a media change 66 h after the drug incubation and allowed the culture to develop for another 24 h into second-generation trophozoite stages. Parasite survival was determined by staining parasites with 2 × SYBR Green I and 165 nM MitoTracker Deep Red (Invitrogen) and a minimum of 8,000 cells were counted by flow cytometry on an Accuri C6 cytometer. Percentage survival was calculated as the parasitemia in the drug-treated sample divided by the parasitemia in the untreated sample × 100.

*P. cynomolgi* M strain (a non-human primate malaria closely related to the human malaria *P. vivax* and forming dormant liver stages) was used for *in vitro* liver stage drug assays (The M strain BPRC was obtained from Centers for Disease Control and Prevention (CDC, USA). Liver stage drug assays were performed in primary rhesus monkey hepatocytes as described^41^. Read out of the assay was performed using a high-content imaging system (Operetta) and analysed with Harmony software. As described previously^42^ two populations of liver stage parasite can be discriminated in 6-day-old cultures: large forms define liver schizonts and persistent small forms are dormant liver stages (hypnozoites). Small forms are defined as having a maximum parasite area of 30 μm^2^.

*Ex vivo P. falciparum* and *P. vivax* drug sensitivity experiments were carried out in Papua Indonesia, an area with documented multidrug-resistant *P. falciparum* and CQ-resistant *P. vivax*., using a modified schizont maturation assay as described previously^13^,^14^,^15^,^16^,^17^. In brief, 200 μl of a 2% haematocrit blood media mixture (BMM), consisting of RPMI-1640 medium plus 10% AB^+^ human serum (*P. falciparum*) or McCoy's 5A medium plus 20% AB^+^ human serum (*P. vivax*) was added to each well of pre-dosed drug plates containing 11 serial concentrations (2-fold dilutions) of the anti-malarials being tested. A candle jar was used to mature the parasites at 37°C for 35–56 h. Incubation was stopped when >40% of ring-stage parasites had reached mature schizont stage in the drug-free control wells. Thick blood films made from each well were stained with 5% Giemsa solution for 30 min and examined microscopically. The number of schizonts per 200 asexual stage parasites was determined for each drug concentration and normalized to the control well. The dose-response data were analysed using nonlinear regression analysis (WinNonLn 4.1, Pharsight) and the IC_50_ value derived using an inhibitory sigmoid Emax model.

Ethical approval for the *ex vivo P. falciparum* and *P. vivax* efficacy data was obtained from the Human Research Ethics Committee of the NT Department of Health & Families and Menzies School of Health Research, Darwin, Australia (HREC 2010-1396) and the Eijkman Institute Research Ethics Commission, Jakarta, Indonesia (EIREC 47 and EIREC 67). Written informed consent was obtained from all patients participating in the study.

The SMFA study was conducted as previously described where mature gametocytes were incubated for 24 h with different concentrations of E209 prior to performing mosquito feeds^43^,^44^,^45^. The RSA_0–3 h_ was performed as described in Witkowski *et al*.,^21^ whereby tightly synchronized early ring-stage parasites were exposed to a drug pulse for 6 h at 700 nM. After drug removal (by washing) the parasites were returned to culture. Parasitemias were then assessed 66 h later on thin blood smears by two independent microscopists and the percentage survival was assessed in comparison to parasites not exposed to drug, as described by Straimer *et al*.^23^ Assays were performed in duplicate on three separate occasions. Parasite reduction ratios were conducted as described in Supplementary Methods^20^.

### *In vivo* efficacy

Studies of murine *P. falciparum* infection were ethically reviewed and carried out in accordance with European Directive 2010/63/EU and the GSK Policy on the Care, Welfare and Treatment of Laboratory Animals. *In vivo* efficacy against *P. falciparum* was conducted^25^ in age-matched female immunodeficient NOD.Cg-*Prkdcscid Il2rgtm1Wjl*/SzJ mice (8–10 weeks of age; 22–24 g) supplied by Charles River, UK, under licence of The Jackson Laboratory, Bar Harbor. Mice were engrafted with human erythrocytes (Red Cross Transfusion Blood Bank in Madrid, Spain) by daily intraperitoneal injection with 1 ml of a 50% haematocrit erythrocyte suspension (RPMI-1640 (Invitrogen), 25 mM HEPES (Sigma), 25% decomplemented AB+ human serum (Sigma) and 3.1 mM hypoxanthine (Sigma)). Mice with ∼40% circulating human erythrocytes were intravenously infected with 2 × 10^7^ *P. falciparum* Pf3D7^0087/N9^-infected erythrocytes (day 0). Efficacy was assessed by administering one oral dose of E209 (2.5, 5, 15, 30, 50 and 100 mg kg^−1^) at day 3 after infection. Treatment group assignments were allocated randomly. Parasitemia was measured by flow cytometry in samples of peripheral blood stained with the fluorescent nucleic acid dye SYTO-16 (Molecular Probes) and anti-murine erythrocyte TER119 monoclonal antibody (Becton Dickinson) in serial 2 μl blood samples taken every 24 h until assay completion. The ED90 was estimated by fitting a four parameter logistic equation using GraphPad 6.0 Software.

### Systemic exposure measurement in infected *Pf* SCID mice

The levels of E209 were evaluated in whole blood to determine standard PK parameters in the individual animals used in the efficacy study. Peripheral blood samples (5 μg ml^−1^) were taken at different times (0.25, 0.5, 1, 2, 4, 6, 8 and 23 h) after drug administration, mixed with 25 μl of Milli-Q water and immediately frozen on dry ice. The frozen samples were stored at −80 °C until analysis. Vehicle-treated mice experienced the same blood-sampling regimen. Blood samples were processed by liquid–liquid extraction. Quantitative analysis by Liquid chromatography-tandem mass spectrometry (LC–MS/MS) was performed using a Waters UPLC system and Sciex API4000 mass spectrometer. The lower limit of quantification in this assay was 5 ng ml^−1^. Blood concentration versus time was analysed by non-compartmental analysis (NCA) using Phoenix ver.6.3 (from Pharsight), from which exposure-related values (*C*_max_ and AUC_0–23_, AUC_0–*t*_) and *t*_max_ were estimated.

### *In vitro* and *in vivo* PK studies

Details of the *in vitro* PK analysis of E209 are contained in Supplementary Tables 1, 2, 3, 17, 19 and 19). *In vitro* metabolic stability of E209 was carried out as described in Supplementary Methods.

*In vivo* PK studies in rats and mice were conducted using established procedures in accordance with the Australian Code of Practice for the Care and Use of Animals for Scientific Purposes, and the study protocols were reviewed and approved by the Monash Institute of Pharmaceutical Sciences Animal Ethics Committee.

Rat PK studies were conducted in fasted 7–9-week-old male Sprague–Dawley rats, as previously described by Charman *et al*.^12^ For intravenous administration E209 was dissolved in 5% glucose solution under sonication (for 10 min) prior to addition of Tween 80 and further sonication (5 min) to produce a clear solution. The formulation was filtered through a 0.22 μm syringe filter prior to dosing and aliquots retained to determine the dose administered. E209 was administered intravenously as a 10 min constant rate infusion via an indwelling jugular vein cannula (1 ml per rat, *n*=2 rats).

For oral dosing, E209 was dissolved in 0.5% [w/v] hydroxypropyl methylcellulose containing 0.4% [w/v] Tween 80 and 0.5% [v/v] benzyl alcohol and sonicated for 5 min producing a clear solution. The bulk formulation was mixed by inverting the tubes prior to drawing each dosing volume. Aliquots of the dosing solution were retained for analysis to determine the dose administered. After the oral formulation (1 ml per rat, *n*=2 rats) was administered, an additional volume (1 ml) of Milli-Q water was administered (via a separate syringe).

Blood samples were collected using a Culex automated blood sampler over 48 h post-dosing into tubes containing anticoagulant and stabilization cocktail (Complete, potassium fluoride and EDTA) to minimize the potential for *ex vivo* degradation and immediately centrifuged at 4 °C. Plasma was separated, stored at −20 °C until processing and assayed by LC/MS using either a Waters Micromass Quattro Ultima PT triple quadrupole instrument coupled to a Waters 2795 HPLC or a Waters Micromass Quattro Premier triple quadrupole instrument coupled to a Waters Acquity UPLC.

The PKs of E209 was also studied in non-fasted 6-8 week old female Swiss Outbred mice that had access to food and water *ad libitum* throughout the pre- and post-dose sampling period. The formulations for intravenous and oral administration were the same as used for the rat studies (study protocols were reviewed and approved by the Monash Institute of Pharmaceutical Sciences Animal Ethics Committee).

E209 was administered intravenously by bolus tail vein injection (50 μl per mouse) or orally by gavage (10 ml kg^−1^) and blood samples collected over 30 h post-dosing (*n*=2 mice per time point). A maximum of two samples were obtained from each mouse, with samples being taken either via submandibular bleed (conscious sampling) or terminal cardiac puncture (underinhaled isofluorane anaesthesia). Blood was collected into tubes in the same way as described for the rat studies and immediately centrifuged and stored at −20 °C until analysis by LC–MS.

Plasma PK parameters were calculated based on compartmental PK analysis using Pmetrics^46^ as described in Supplementary Methods. A classical three compartment oral absorption model was used for all tested species due to the tri-phasic profiles displayed over time. For human predictions, allometric scaling was performed based on weight. Clearance rates (including inter-compartmental clearance rates Q1 and Q2) were predicted according to the following equations:


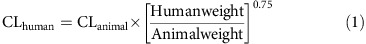



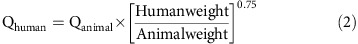


Other PK parameters were scaled linearly and simulation of human exposure to TDD-E209 was performed assuming a 15 mg kg^−1^ dose using Pmetrics where median values were plotted over time on a semi-log scale.

### hERG profiling and assessment of cytotoxicity

The potencies (IC_50_ values) for E209 to inhibit the cardiac I_Kr_ (hERG), Cav_1.2_ and Nav_1.5_ channels were determined in an electrophysiology-based assays using IonWorks HT (CHO cells)^27^.

### *In vivo* (rat) safety studies

A single, rising oral dose study to determine the MTD of E209 and aid in the selection of dose levels for the 7-day toxicity study. The rats (Sprague–Dawley, age 6–7 weeks) were divided into four groups, each with three male and three female. Dose levels employed were 30, 100, 300 and 1,000 mg kg^−1^ and the MTD was shown to be 300mg kg^−1^.

Following this single dose MTD study, rats were dosed once a day at 0, 30, 100 and 300 mg kg^−1^ po E209 for 7 days. E209 was formulated in 0.5 % [w/v] HPMC, 0.4% Tween 80 [w/v], 0.5% [v/v] benzyl alcohol in purified water. In each group there were eight Sprague–Dawley rats of each sex. At the end of the dosing period, five rats/sex/group were killed (day 8) and the remaining three rats/sex/group were left for recovery. In addition, six rats of each sex per group were allocated for toxicokinetics.

*In vivo* PK studies conformed to AAALAC International and NIH guidelines as reported in the Guide for the Care and Use of Laboratory Animals, National Research Council (2011); People's Republic of China, Ministry of Science & Technology, ‘Regulations for the Administration of Affairs Concerning Experimental Animals', 1988.

### Data availability

All relevant data are available from the authors upon request.

## Additional information

**How to cite this article:** O'Neill, P. M. *et al*. A tetraoxane-based antimalarial drug candidate that overcomes PfK13-C580Y dependent artemisinin resistance. *Nat. Commun.* **8,** 15159 doi: 10.1038/ncomms15159 (2017).

**Publisher's note:** Springer Nature remains neutral with regard to jurisdictional claims in published maps and institutional affiliations.

## Supplementary Material

Supplementary Information Supplementary Figures, Supplementary Tables, Supplementary Notes, Supplementary Methods and Supplementary References

## Figures and Tables

**Table 1:** Calculated physicochemical* properties and *in vitro* and *in vivo* antimalarial activity† profiles of selected tetraoxane derivatives.

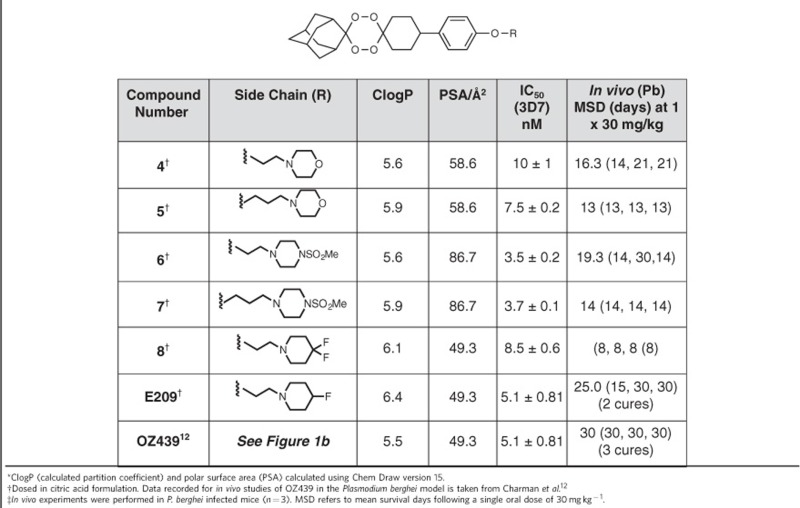					

**Table 2:** *In vivo* and *in vitro* pharmacokinetic (PK) parameters for E209 as predicted using compartmental PK analysis in male Beagle dogs, female Swiss outbred mice and the male Sprague–Dawley rat following IV and oral administration.

	**Rat (mean)**	**Mouse (mean)**	Dog* **(mean)**	**Human (mean)**
*In vivo PK parameters*				
IV dose (mg kg^−1^)	2	2	1	N/A
PO dose (mg kg^−1^)	9	10	5	15mg†
Central clearance, CL (l^ ^h^−1^ kg^−1^)	1.6	4.3	1.1	0.41
Central volume of distribution *V*_c_ (l kg^−1^)	0.54	0.99	0.76	0.54†
Inter-compartmental clearance 1, Q_1_ (l ^−1 ^h^−1^ kg^−1^)	1.76	10.3	2.8	0.45†
Inter-compartmental clearance 2, Q_2_ (l h^−1^ kg^−1^)	0.08	0.97	0.13	0.020
Peripheral Volume of distribution 1, *V*_p1_ (l kg^−1^)	2.1	1.49	0.37	2.1†
Peripheral volume of distribution 2, *V*_p2_ (l kg^−1^)	2.0	10.8	2.95	2.0†
Absorption rate constant *K*_a_ (h^−1^)	0.31	0.79	0.33	0.31†
*F* (%)	62	82	40	62†
				
*In vitro microsomal data*				
Degradation half-life (min)	48	132	173	68
*In vitro* CL_int_ (μl min^−1^ mg^−1^ protein)	36	13	10	25
Microsome-predicted *E*_H_	0.48	0.22	0.38	0.50

^*^Supplementary Methods and Supplementary Table 9 in Supporting Information.

^†^On the basis of allometric scaling from rat parameters.
