# Supplementary material for: A tetraoxane-based antimalarial drug candidate that overcomes PfK13-C580Y dependent artemisinin resistance
Source: Nat Commun. 2017 May 24;8:15159. doi: 10.1038/ncomms15159 (PMC5458052; doi:10.1038/ncomms15159)
Supplement: Supplementary Information — Supplementary Figures, Supplementary Tables, Supplementary Notes, Supplementary Methods and Supplementary References [file ncomms15159-s1.pdf]

Supplementary Figures

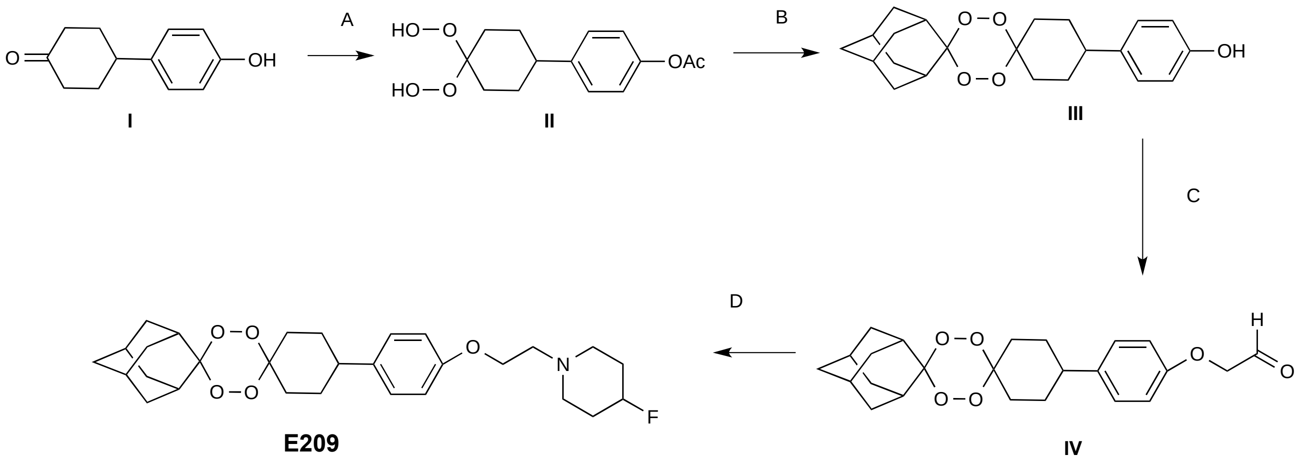

Supplementary Figure 1: Synthesis of Tetraoxane E209 (A)(i) acetyl chloride, Et_3N, dichloromethane, 0^oC, 3 hours (ii) 30 %H_2O_2, HCO_2H/acetonitrile=1:1 0° C-room temperature, 1hour (B)(i) 2-adamantanone, $Bi(OTf)_3$ dichloromethane, room temperature, 1 hour (ii) MeOH, THF, KOH, 50 ^oC, 2-3 hours (C)(i) allyl bromide, K_2CO_3, acetone,reflux o/n (ii) O_3, DCM/MeOH; Ph_3P (D) $NaBH(OAc)_3$,4-fluoropiperidine, dichloromethane, room temperature

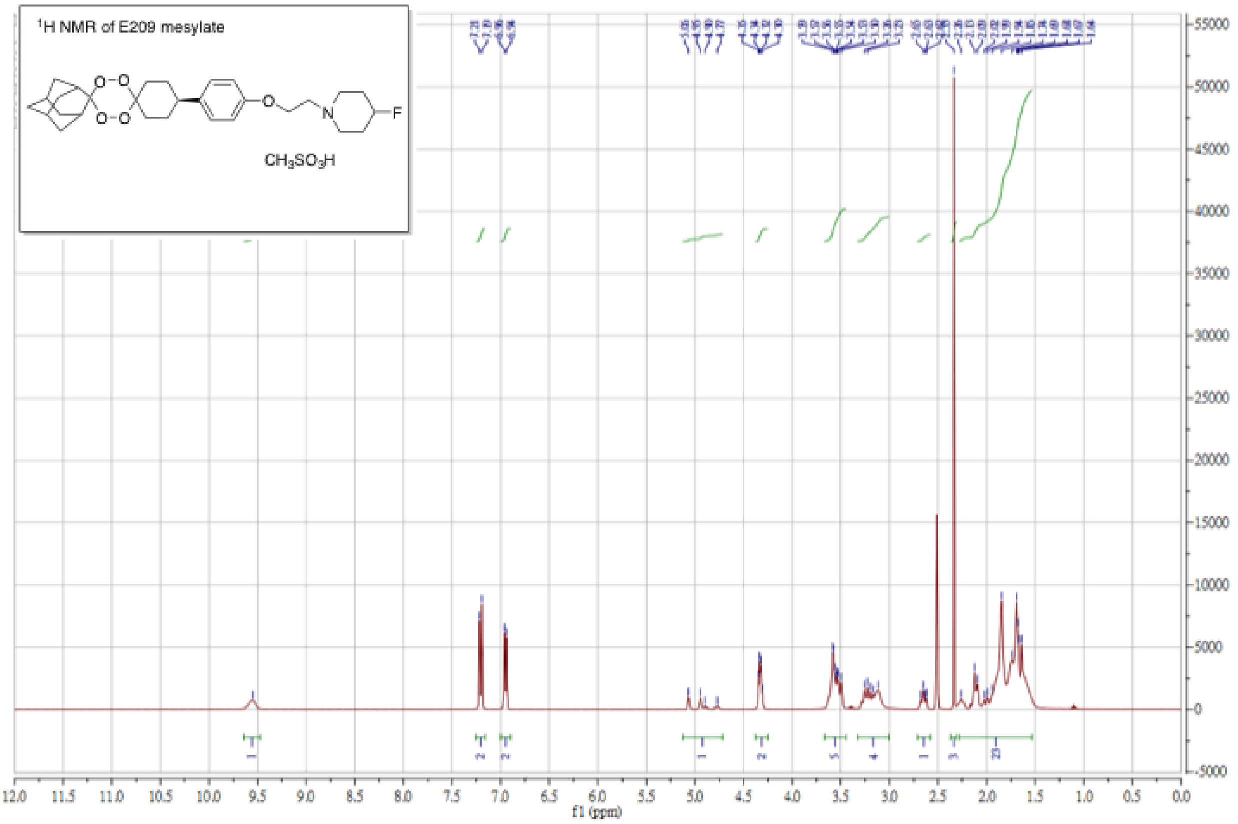

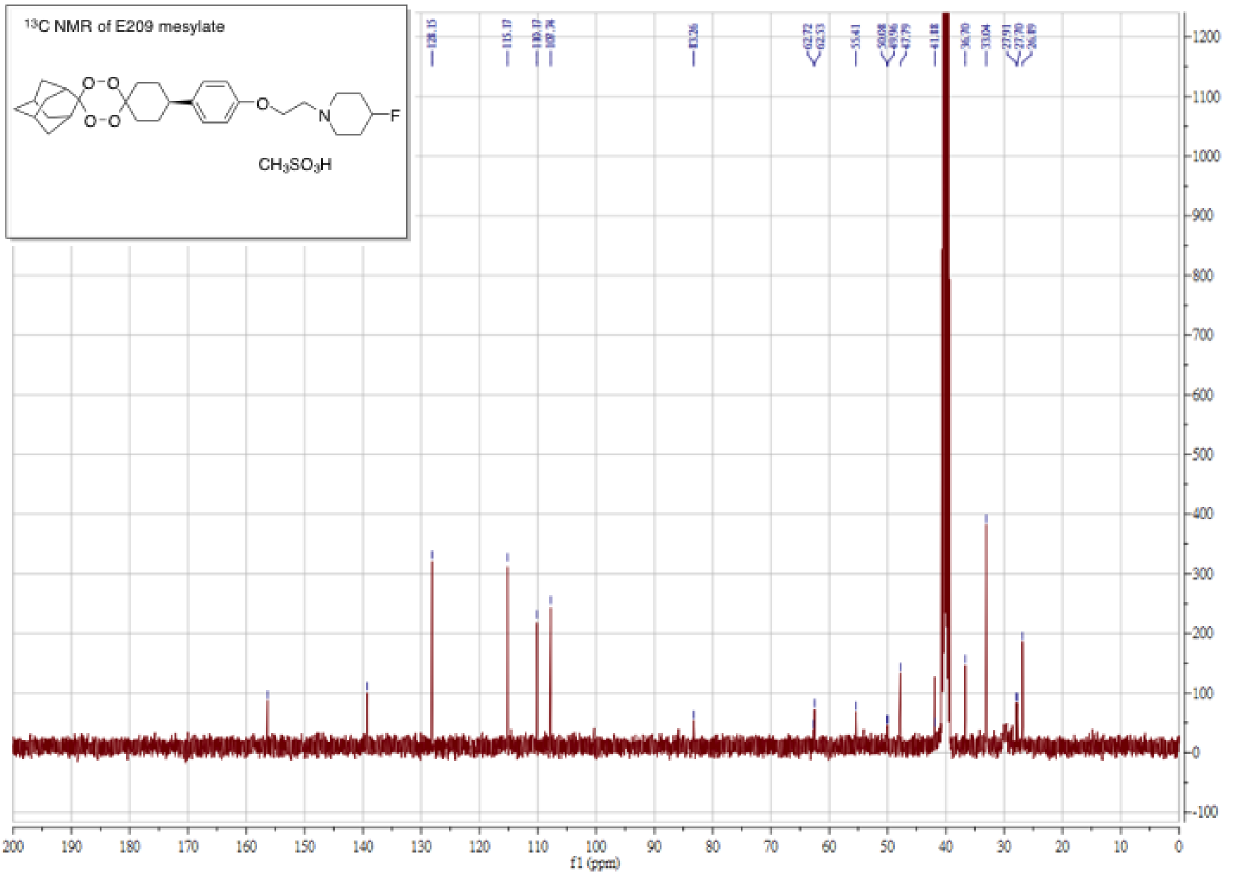

Supplementary Figure 2: ^{1}H NMR and ^{13}C NMR Spectra for E209 mesylate salt.

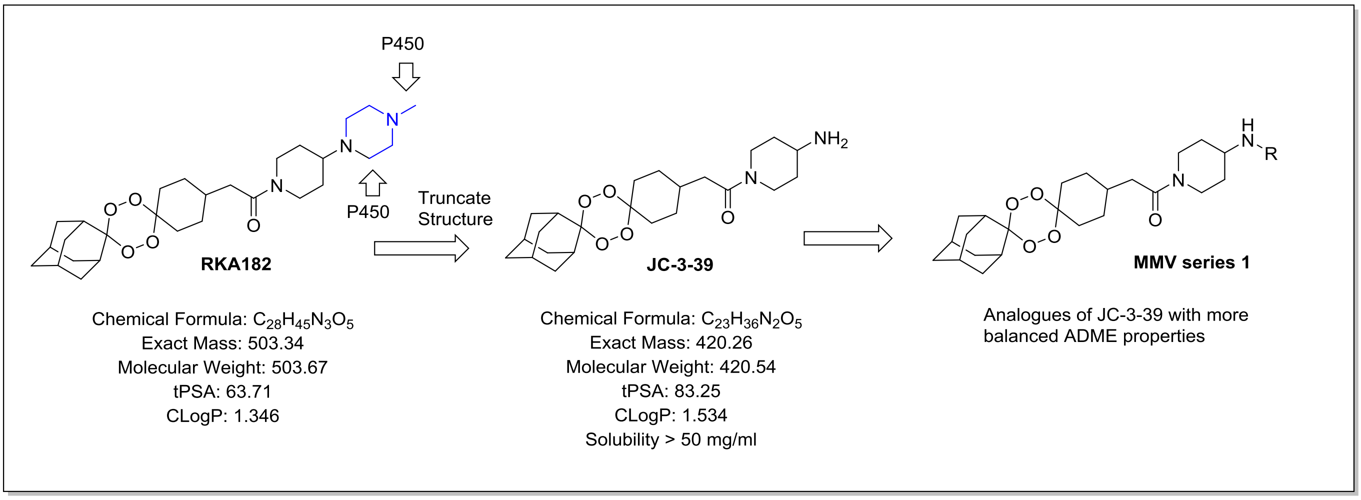

Supplementary Figure 3: Initial Medicinal Chemistry led Optimisation Studies from JC-3-39

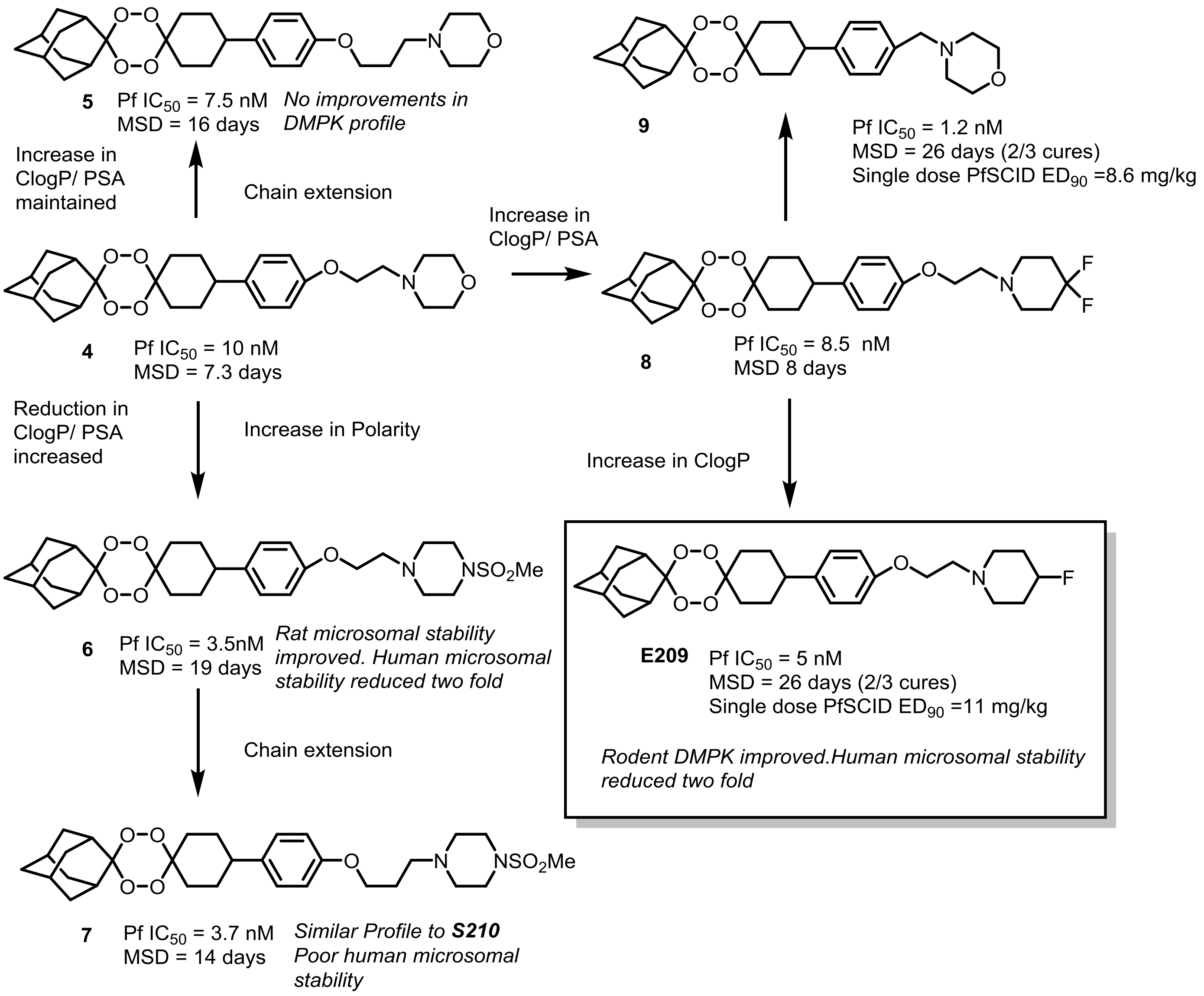

Supplementary Figure 4: Medicinal Chemistry Optimisation Studies of MMV Series 2 towards E209

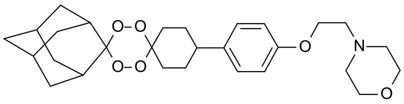	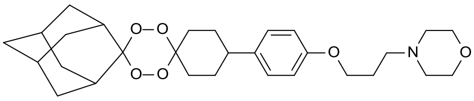
**NR-2-24 (4)**	**S201 (5)**
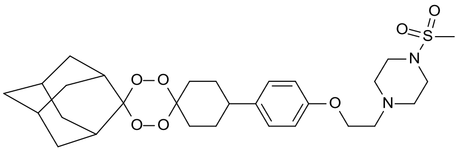	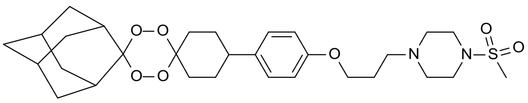
**S204 (6)**	**A217 (7)**
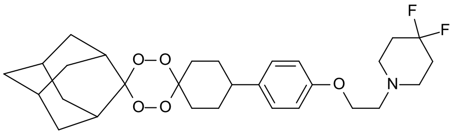	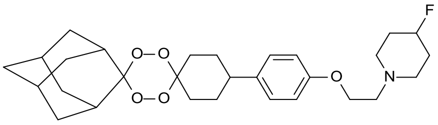
**E207 (8)**	**E209**
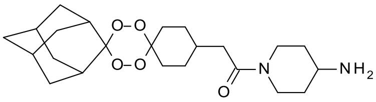	
**JC-3-39**	

Supplementary Figure 5: Structures of tetraoxanes evaluated in Blood Stability Studies

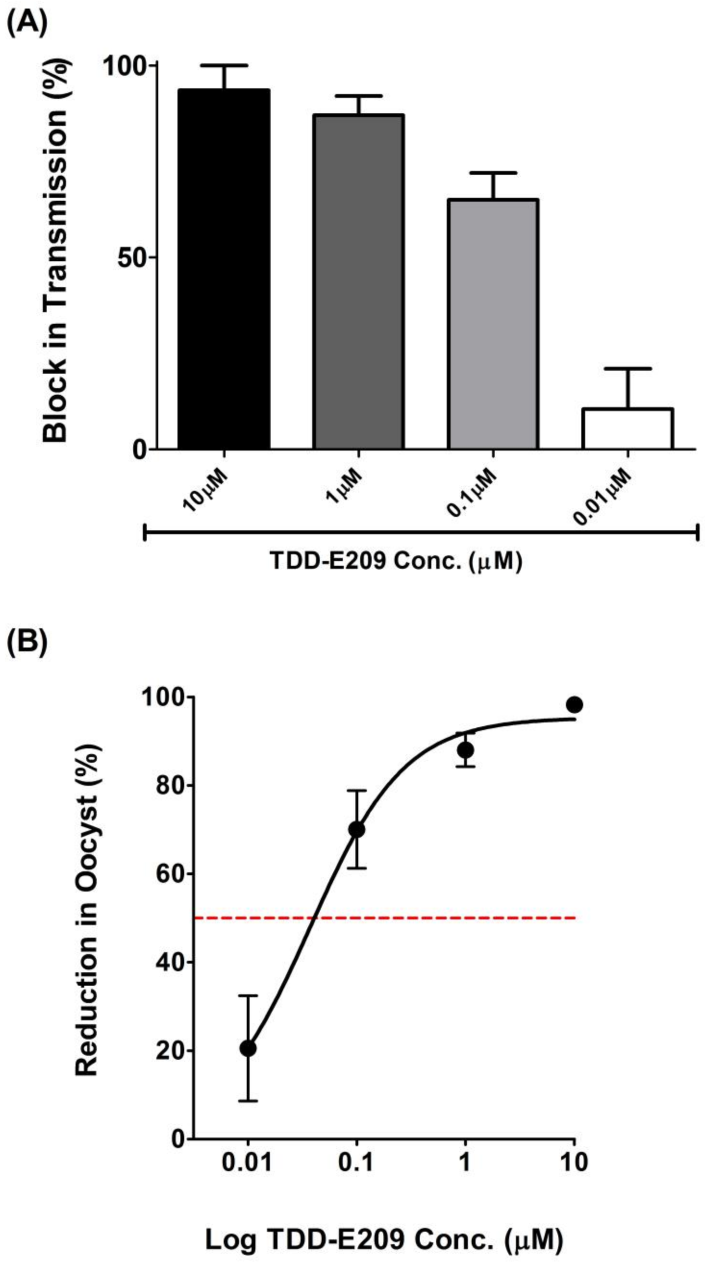

Supplementary Figure 6: *Plasmodium falciparum* Transmission Blocking Activity of E209 in SMFA

(A) Percent block in transmission denotes the % of mosquitoes in which no oocysts were observed after infection with drug treated gametocytes normalised to the non-drug-treated DMSO control group. ; (B) Relationship between E209 concentration (µM) and reduction in oocyst intensity (%), Data represent average values and SE, generated from two separate experiments containing a total of 39 different replicates (each replicate representing a single mosquito). The red dashed line represents 50% reduction in mean oocyst intensity.

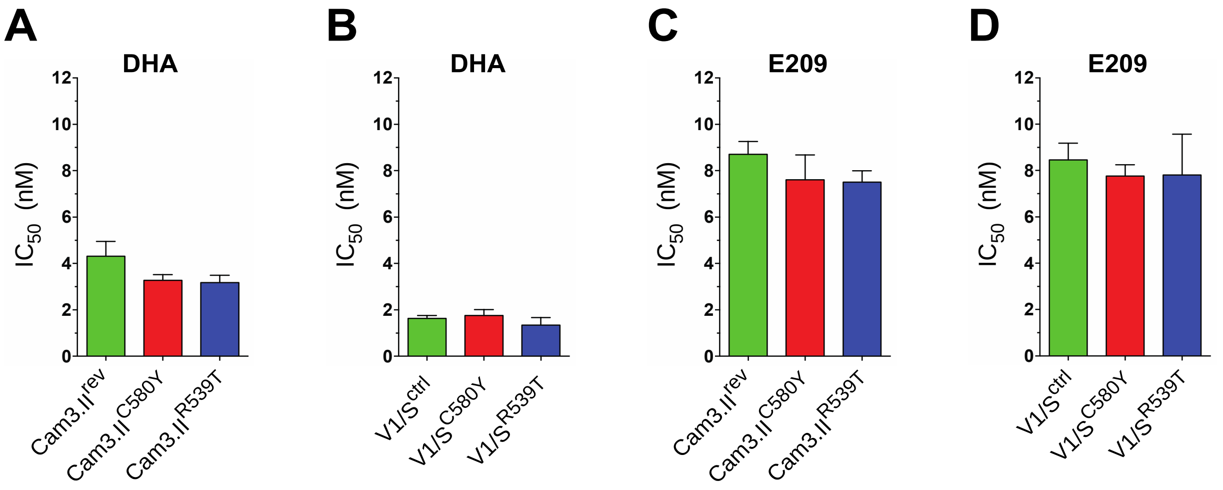

Supplementary Figure 7: *In vitro* proliferation assays show no significant differences in IC50 values between K13 wild-type and mutant parasites. IC50 values (mean±SEM) were measured in 72-h proliferation assays with final parasitemias determined using flow cytometry with SYBR Green I and MitroTracker Deep Red-stained parasites. Assays were performed on three separate occasions in duplicate. DHA, dihydroartemisinin, E209.

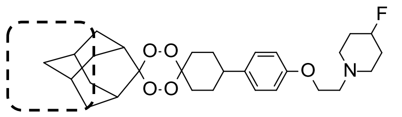

M+16 (I), (II) & (III), M+32 (I)

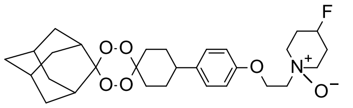

M+16 (IV)

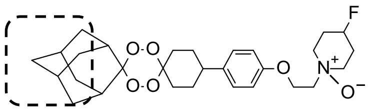

M+32 (II) & (III)

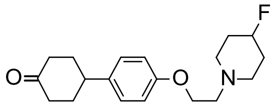

M-182

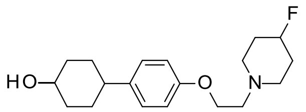

M-180

Supplementary Figure 8: Structures of putative metabolites for E209. Dotted line indicates proposed site of hydroxylation.

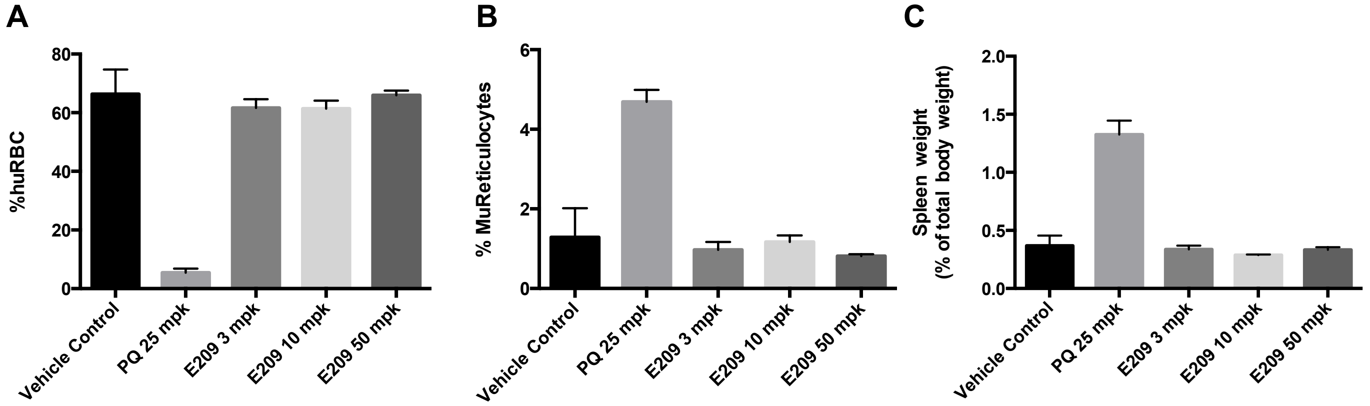

Supplementary Figure 9. Assessment of haemolytic toxicity in G6PD deficient mice. NOD/SCID mice engrafted with A- variant G6PD deficient huRBC were treated with PQ at 25 mg/kg/day for 3 days as a positive control for haemolytic toxicity, with vehicle control for 3 days, or with E209 at 3, 10 or 50 mpk for 4 days. The percent of human (hu)RBC (Glycophorin A+ cells) (A) or percent of murine (mu)reticulocytes (CD71+Ter199+) (B) in circulation at 7 days post-initiation of treatment was measured by flow cytometry. At termination of experiment at day 7, spleen weights were also measured (C) and were expressed as a percent of total body weight. n=4-5.

Supplementary Tables

Supplementary Table 1: A comparison of key *in vitro* and *in vivo* potency data with DMPK properties

Compound Number	*In vitro* CL_{int} (ml/min/Kg) H/R[a]	Rat PK iv/ CL(ml/min/kg)[a] /$T_{1/2}$(h)	Rat Blood Stability/*in vitro* $T_{1/2}$ (h)
**4**	135/86	7.1/1.1	14
**5**	147/75	6.5/1.5	15
**6**	257/38	3.5/3.2	12
**7**	238/29	3.5/3.2	15
**8**	107/60	1.3/2.8	16
**E209**	58/70	5.1/4.0	13

[a] Data generated by Chem Partners; CLint = the intrinsic clearance in in vitro studies in human (H) and rat (R) liver microsomes; **iv/ CL(ml/min/kg),** intravenous (iv) clearance following a 1 mg/kg dose in rats.

Supplementary Table 2: Metabolic stability parameters for N205 and E209 based on NADPH-dependent degradation profiles in human, rat and mouse liver microsomes.

**Compound Details**	**Species**	**Substrate Concentration (μM)**	**Degradation half-life** (min)	***In vitro*** **CL_{int}** (μL/min/mg protein)	**Microsome-Predicted E_H**
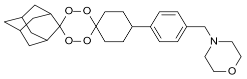 **N205**	Human	1	34 (33, 35)	51 (53, 50)	0.67 (0.68, 0.66)
		5	43 (44, 41)	41 (39, 42)	0.62 (0.61, 0.63)
	Rat	1	15 (13, 16)	118 (130, 105)	0.75 (0.77, 0.73)
		5	20 (17, 23)	89 (101, 76)	0.69 (0.72, 0.66)
	Mouse	1	48 (42, 54)	37 (41, 32)	0.44 (0.47, 0.41)
		5	87 (93, 82)	20 (19, 21)	0.30 (0.29, 0.31)
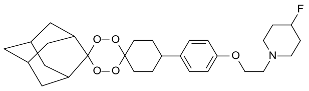 **E209**	Human	1	68 (68, 69)	25 (26, 25)	0.50 (0.50, 0.50)
		5	76 (89, 64)	23 (20, 27)	0.48 (0.44, 0.52)
	Rat	1	48 (46, 50)	36 (38, 34)	0.48 (0.49, 0.47)
		5	54 (57, 51)	32 (30, 34)	0.45 (0.44, 0.46)
	Mouse	1	132 (122, 143)	13 (14, 12)	0.22 (0.23, 0.21)
		5	149 (233, 65)	17 (7, 27)	0.25 (0.14, 0.36)

Supplementary Table 3: Solubility data for tetraoxanes at 37°C over a 6 hour incubation (mean of three measurements). All solubility values refer to the free base equivalent.

Compound	Media	Media pH	pH at equilibrium	Solubility (µg/mL)		
				1 h	4 h	6 h
E209 mesylate	0.1 N HCl	1.0	0.8	46.1	48.9	46.3
	0.01 N HCl	2.0	1.9	> 2000[a,b]	> 2000[b]	>2000[b]
	FeSSIF	5.0	5.0	3355	4495	3870
	FeSSIF blank		5.0	4.0	2.7	3.5
	FaSSIF	6.5	6.3	117	132	121
	FaSSIF blank		6.3	< 0.1	< 0.1	< 0.1
	Phosphate buffer	7.4	7.4	< 0.1	< 0.1	< 0.1
N205 mesylate	0.1 N HCl	1.0	1.0	21.2	17.2	16.0
	0.01 N HCl	2.0	1.9	250	172	155
	FeSSIF	5.0	4.7	1089	755	783
	FeSSIF blank		4.9	2.3	0.9	1.2
	FaSSIF	6.5	6.1	24.0	18.1	26.7
	FaSSIF blank		6.3	< 0.1	< 0.1	< 0.1
	Phosphate buffer	7.4	7.4	< 0.1	< 0.1	< 0.1

> indicates that the medium was not saturated when the measurement was taken;
< indicates values below the quantitation limit of the method
[a] Formed a gel prior to complete solubilisation
[b] Sample appeared clear indicating that it was not saturated
"not meas." indicates a measurement was not taken

Supplementary Table 4: *In vitro* IC_{50} values for E209 against several sensitive and resistant strains of the malarial parasite. *P. falciparum* strains included in the study were obtained from the Malaria Research and Reference Reagent Resource Center (MR4;www.mr4.org).[4]

Parasite strain	E209 IC_{50} (nM)	Artesunate	Chloroquine
***NF54**	5.2 ± 1.0	4.7 ± 1.0	5.9 ± 1.0
**#K1**	4.2 ± 0.8	2.8 ± 0.6	173.0 ± 29.5
***HB3**	4.2 ± 0.8	2.8 ± 0.6	9.0 ± 1.5
**#7G8**	2.9 ± 0.6	1.8 ± 0.4	55.0 ± 9.4
**#TM90C2B**	7.3 ± 1.4	3.8 ± 0.8	121.0 ± 20.6
***D6**	8.5 ±1.6	5.8 ± 1.2	8.6 ± 1.5
**#V1/S**	4.2 ± 0.8	2.7 ± 0.6	193.5 ± 33.0
**#Dd2**	7.7 ± 1.5	5.4 ± 1.1	150.5 ± 25.7
**#FCB**	14.0 ± 2.7	5.8 ± 1.2	66.0 ± 11.3
***3D7**	5.1 ± 0.8		

*****Chloroquine sensitive strains, # Chloroquine resistant strains. ART = artesunate Values are means ± SD (*n*=3)

Supplementary Table 5: *Ex-vivo* E209 susceptibility in clinical *P. falciparum* and *P. vivax* isolates.

Species	*P. falciparum* lab lines[1] IC_{50} (nM)		*P. falciparum* clinical field isolates		*P. vivax* clinical field isolates	
**Drug**	**FC27 (CQ^S)**	**K1 (CQ^R)**	**n[2] (%)**	**Median IC_{50} (range), nM**	**n[2] (%)**	**Median IC_{50} (range), nM**
Chloroquine	32.3	212.2	9 (100)	85.5 (35.6-129.2) ***p*=0.008**	12 (92)[3]	146.6 (13.4-254.6) ***p*=0.002**
Amodiaquine	33.1	39.3	9 (100)	25.6 (17.9-41.9) ***p*=0.021**	13 (100)	50.6 (9.6-91.6) ***p*=0.004**
Piperaquine	54.6	75.5	9 (100)	35.6 (20.2-56.8) ***p*=0.008**	12 (92)[3]	33.8 (8.9-121.3) ***p*=0.015**
Mefloquine	56.2	20.0	8 (89)[4]	12.7 (3.4-33.8) *p*=0.327	12 (92)[3]	18.0 (7.7-51.5) *p*=0.272
Artesunate	3.6	5.1	8 (89)[5]	2.4 (0.6-7.5) *p*=0.012	12 (92)[3]	0.8 (0.3-5.7) *p*=0.002
**TDD-E209**	**20.7**	**31.8**	**9 (100)**	**15.7 (3.1-48.2)**	**13 (100)**	**10.5 (4.5-77.4)**

[1] Median IC_{50} values (derived from 3 independent experiments) assessed by *in vitro* schizont maturation quantified by microscopy
CQ^S, chloroquine sensitive laboratory strain
CQ^R, chloroquine resistant laboratory strain
[2] Total number of assays with acceptable IC_{50} (percentage of samples which fulfilled criteria for successful culture)
[3] One drug plate containing CQ, PIP, MFQ, and AS contaminated
[4] Insufficient BMM mix to test MFQ for one isolate
[5] Assay abort for AS in one isolate
[#] Comparison with **TDD-E209** (Wilcoxon rank sum test); statistically significant *p* values for **lower TDD-E209 IC_{50}s** are shown in **boldface type**

Supplementary Table 6: Correlation of ex-vivo anti-malarial susceptibilities in P. falciparum and P. vivax clinical field isolates

Anti-malarial combination	*P. falciparum*			*P. vivax*		
	r_s[a]	*p*[a]	df[b]	r_s	*p*	df
TDD-E209-CQ	0.683	**0.042**	9	0.224	0.485	12
TDD-E209-AQ	0.350	0.356	9	-0.077	0.803	13
TDD-E209-PIP	0.550	0.125	9	0.287	0.366	12
TDD-E209-MFQ	0.357	0.385	8	0.455	0.138	12
TDD-E209-AS	-0.024	0.955	8	0.105	0.746	12

[a] Spearman rank correlation; statistically significant values are shown in boldface type

[b] df, degrees of freedom

Supplementary Table 7: Results of two-sample t-test with unequal variances, $RSA_{0\text{-}3h}$ 700nM DHA; V1/S vs V1/S^{C580Y} (groups 1 vs. 2 respectively; $p<0.001$; Figure 3)

```
. ttest logV1SC580Y, by(group) unequal
```

Two-sample t test with unequal variances

Group	Obs	Mean	Std. Err.	Std. Dev.	[95% Conf.	Interval]
1	3	-1.339128	.1713105	.2967185	-2.076217	-.6020383
2	3	2.210186	.1168013	.2023059	1.707631	2.712742
combined	6	.4355293	.7990492	1.957263	-1.618492	2.489551
diff		-3.549314	.2073399		-4.156584	-2.942045

```
    diff = mean(1) - mean(2)                                      t = -17.1183
Ho: diff = 0                     Satterthwaite's degrees of freedom =  3.52904

    Ha: diff < 0                 Ha: diff != 0                 Ha: diff > 0
 Pr(T < t) = 0.0001         Pr(|T| > |t|) = 0.0002          Pr(T > t) = 0.9999
```

Supplementary Table 8: Results of two-sample t-test with unequal variances, $RSA_{0\text{-}3h}$ 700nM DHA; V1/S vs V1/S^{R539T} (groups 1 vs. 2 respectively; p<0.001; Figure 3)

```
. ttest logV1SR539T, by(group) unequal

Two-sample t test with unequal variances
------------------------------------------------------------------------------
   Group |     Obs        Mean    Std. Err.   Std. Dev.   [95% Conf. Interval]
---------+--------------------------------------------------------------------
       1 |       3   -1.339128    .1713105    .2967185   -2.076217   -.6020383
       2 |       2    3.154193    .1308453    .1850432    1.491645     4.81674
---------+--------------------------------------------------------------------
combined |       5    .4582004    1.105401    2.471752   -2.610886    3.527286
---------+--------------------------------------------------------------------
    diff |           -4.493321    .2155639               -5.181498   -3.805143
------------------------------------------------------------------------------
    diff = mean(1) - mean(2)                                      t = -20.8445
Ho: diff = 0                     Satterthwaite's degrees of freedom =  2.98346

    Ha: diff < 0                 Ha: diff != 0                 Ha: diff > 0
 Pr(T < t) = 0.0001         Pr(|T| > |t|) = 0.0003          Pr(T > t) = 0.9999
```

Supplementary Table 9: Results of two-sample t-test with unequal variances, RSA$_{0\text{-}3h}$ 700nM DHA; Cam3.II^{rev} vs Cam3.II^{C580Y} (groups 1 vs. 2 respectively; p<0.01; Figure 3)

```
. ttest logCamC580Y, by(group) unequal

Two-sample t test with unequal variances
------------------------------------------------------------------------------
   Group |     Obs        Mean    Std. Err.   Std. Dev.   [95% Conf. Interval]
---------+--------------------------------------------------------------------
       1 |       4   -.5739938    .2546709    .5093417    -1.38447    .2364826
       2 |       3     1.66302    .0165498    .0286651    1.591812    1.734228
---------+--------------------------------------------------------------------
combined |       7    .3847264    .4720425    1.248907   -.7703199    1.539773
---------+--------------------------------------------------------------------
    diff |           -2.237014     .255208               -3.045369   -1.428659
------------------------------------------------------------------------------
    diff = mean(1) - mean(2)                                      t =  -8.7655
Ho: diff = 0                     Satterthwaite's degrees of freedom =  3.02531

    Ha: diff < 0                 Ha: diff != 0                 Ha: diff > 0
 Pr(T < t) = 0.0015         Pr(|T| > |t|) = 0.0030          Pr(T > t) = 0.9985
```

Supplementary Table 10: Results of two-sample t-test with unequal variances, $RSA_{0\text{-}3h}$ 700nM DHA; Cam3.II^{rev} vs Cam3.II^{R539T} (groups 1 vs. 2 respectively; $p<0.001$; Figure 3)

```
. ttest logCam3, by(group) unequal
```

Two-sample t test with unequal variances

Group	Obs	Mean	Std. Err.	Std. Dev.	[95% Conf.	Interval]
1	4	-.5660566	.2485161	.4970322	-1.356946	.2248325
2	3	3.196533	.0400866	.069432	3.024054	3.369011
combined	7	1.046482	.7718259	2.042059	-.8421084	2.935072
diff		-3.762589	.2517284		-4.541898	-2.983281

```
    diff = mean(1) - mean(2)                                      t = -14.9470
Ho: diff = 0                         Satterthwaite's degrees of freedom =  3.15494

    Ha: diff < 0                 Ha: diff != 0                 Ha: diff > 0
 Pr(T < t) = 0.0002         Pr(|T| > |t|) = 0.0005          Pr(T > t) = 0.9998
```

Supplementary Table 11: Results of two-sample t-test with unequal variances, RSA_{0-3h} 700nM E209; V1/S vs V1/S^{C580Y} (groups 1 vs. 2 respectively; p>0.5; Figure 3)

```
. ttest logV1SC580Y, by(group) unequal

Two-sample t test with unequal variances
```

Group	Obs	Mean	Std. Err.	Std. Dev.	[95% Conf.	Interval]
1	3	1.526395	.1546339	.2678337	.861059	2.191731
2	3	1.582258	.1282524	.2221397	1.030433	2.134084
combined	6	1.554327	.0907089	.2221904	1.321152	1.787501
diff		-.0558634	.2008988		-.6212503	.5095234

```
    diff = mean(1) - mean(2)                                      t =  -0.2781
Ho: diff = 0                     Satterthwaite's degrees of freedom =  3.86776

    Ha: diff < 0                 Ha: diff != 0                 Ha: diff > 0
 Pr(T < t) = 0.3976         Pr(|T| > |t|) = 0.7952          Pr(T > t) = 0.6024
```

Supplementary Table 12: Results of two-sample t-test with unequal variances, $RSA_{0\text{-}3h}$ 700nM E209; V1/S vs V1/S^{R539T} (groups 1 vs. 2 respectively; $p<0.001$; Figure 3)

```
. ttest logV1SR539T, by(group) unequal

Two-sample t test with unequal variances
------------------------------------------------------------------------------
   Group |     Obs        Mean    Std. Err.   Std. Dev.   [95% Conf. Interval]
---------+--------------------------------------------------------------------
       1 |       4    1.476565    .1201617    .2403235    1.094157    1.858973
       2 |       3    2.931516    .1307729    .2265053    2.368846    3.494187
---------+--------------------------------------------------------------------
combined |       7    2.100116    .3049129    .8067237    1.354021    2.846211
---------+--------------------------------------------------------------------
    diff |           -1.454951    .1775961               -1.923294    -.986609
------------------------------------------------------------------------------
    diff = mean(1) - mean(2)                                      t =  -8.1925
Ho: diff = 0                     Satterthwaite's degrees of freedom =  4.61141

    Ha: diff < 0                 Ha: diff != 0                 Ha: diff > 0
 Pr(T < t) = 0.0003         Pr(|T| > |t|) = 0.0006          Pr(T > t) = 0.9997
```

Supplementary Table 13: Results of two-sample t-test with unequal variances, $RSA_{0\text{-}3h}$ 700nM E209; Cam3.II^{rev} vs Cam3.II^{C580Y} (groups 1 vs. 2 respectively; p>0.5; Figure 3)

```
. ttest logCam3C580Y, by(group) unequal

Two-sample t test with unequal variances
```

Group	Obs	Mean	Std. Err.	Std. Dev.	[95% Conf.	Interval]
1	4	2.723721	.1917932	.3835864	2.113349	3.334092
2	5	2.753967	.158434	.3542692	2.314084	3.19385
combined	9	2.740524	.1145932	.3437796	2.476272	3.004777
diff		-.030246	.2487689		-.6321487	.5716567

```
    diff = mean(1) - mean(2)                                      t =  -0.1216
Ho: diff = 0                     Satterthwaite's degrees of freedom =  6.29338

    Ha: diff < 0                 Ha: diff != 0                 Ha: diff > 0
 Pr(T < t) = 0.4535         Pr(|T| > |t|) = 0.9070          Pr(T > t) = 0.5465
```

Supplementary Table 14: Results of two-sample t-test with unequal variances, $RSA_{0\text{-}3h}$ 700nM E209; $Cam3.II^{rev}$ vs $Cam3.II^{R539T}$ (groups 1 vs. 2 respectively; $p<0.05$; Figure 3)

```
. ttest logCam3R539T, by(group) unequal

Two-sample t test with unequal variances
```

Group	Obs	Mean	Std. Err.	Std. Dev.	[95% Conf.	Interval]
1	4	2.723721	.1917932	.3835864	2.113349	3.334092
2	5	3.339173	.1300469	.2907938	2.978105	3.700241
combined	9	3.065639	.1500646	.4501939	2.719589	3.411688
diff		-.6154523	.2317258		-1.194692	-.0362126

```
    diff = mean(1) - mean(2)                                      t =  -2.6560
Ho: diff = 0                     Satterthwaite's degrees of freedom =  5.51793

    Ha: diff < 0                 Ha: diff != 0                 Ha: diff > 0
 Pr(T < t) = 0.0205         Pr(|T| > |t|) = 0.0409          Pr(T > t) = 0.9795
```

Supplementary Table 15: Ring-stage survival assay ($RSA_{0\text{-}3h}$) and fold change in K13 mutant lines when exposed to 700nM DHA, E209 or JC3-39

Parasite	700nM DHA					700nM E209					700nM JC3-39				
	$RSA_{0\text{-}3h}$ Mean	SEM	n	p value[a]	Fold change[b]	$RSA_{0\text{-}3h}$ Mean	SEM	n	p value[a]	Fold change[b]	$RSA_{0\text{-}3h}$ Mean	SEM	n	p value[a]	Fold change[b]
Cam3.II[rev]	0.62	0.14	4			16.07	2.89	4			21.26	4.17	4		
Cam3.II[C580Y]	5.66	0.33	5	0.003	9.1	16.51	2.61	5	0.9070	1.0	21.16	2.36	4	0.887	1.0
Cam3.II[R539T]	24.49	0.97	3	0.0005	39.5	29.14	3.65	5	0.0409	1.8	30.04	6.36	5	0.4153	1.4
V1/S[ctrl]	0.20	0.08	4			4.47	0.53	4			7.49	1.28	3		
V1/S[C580Y]	9.24	1.05	3	0.0002	46.2	4.94	0.59	3	0.7952	1.1	7.83	1.63	3	0.9392	1.0
V1/S[R539T]	23.64	3.08	2	0.0003	118.2	19.07	2.42	3	0.0006	4.3	23.87	2.70	3	0.0104	3.2

[a] *p value* were calculated with a two-sample t-test with unequal variances and compared to the respective reference line Cam3.II[rev] and V1/S[ctrl]

[b] increase of $RSA_{0\text{-}3h}$ compared to the respective reference line Cam3.II[rev] and V1/S[ctrl]

SEM: Standard error of the mean; n=number of repeats (assay)

Supplementary Table 16: IC_{50} data for DHA, E209 and JC3-39

Parasite	IC_{50} DHA			IC_{50} E209			IC_{50} JC3-39		
	IC_{50} Mean (nM)	SEM	n	IC_{50} Mean (nM)	SEM	n	IC_{50} Mean (nM)	SEM	n
Cam3.II^{rev}	4.31	0.65	5	8.71	0.55	3	2.43	0.56	3
Cam3.II^{C580Y}	3.28	0.25	4	7.60	1.08	3	2.21	0.36	3
Cam3.II^{R539T}	3.18	0.31	3	7.51	0.48	3	1.82	0.06	3
V1/S^{ctrl}	1.63	0.13	3	8.46	0.72	3	2.58	0.33	3
V1/S^{C580Y}	1.77	0.25	3	7.76	0.49	3	2.42	0.39	3
V1/S^{R539T}	1.34	0.33	3	7.81	1.76	3	2.10	0.60	3

SEM: Standard error of the mean; n=number of repeats (assay)

Supplementary Table 17: Metabolic stability parameters for E209 based on NADPH-dependent degradation profiles in human, dog, rat and mouse liver microsomes.

Compound Details	Species	Substrate Concentration (µM)	Degradation half-life (min)	*In vitro* CL_{int} (µL/min/mg protein)	Microsome-Predicted E_H
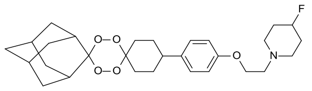 **E209**	Human	1	68 (68, 69)	25 (26, 25)	0.50 (0.50, 0.50)
		5	76 (89, 64)	23 (20, 27)	0.48 (0.44, 0.52)
	Dog	1	173	10	0.38
	Rat	1	48 (46, 50)	36 (38, 34)	0.48 (0.49, 0.47)
		5	54 (57, 51)	32 (30, 34)	0.45 (0.44, 0.46)
	Mouse	1	132 (122, 143)	13 (14, 12)	0.22 (0.23, 0.21)
		5	149 (233, 65)	17 (7, 27)	0.25 (0.14, 0.36)

Data represent the mean values of two replicate measurements. In the case of dog microsomes, data are for n=1 only and the study was only conducted at a single incubation concentration.

Supplementary Table 18: List of E209 metabolites monitored for in human, rat and mouse liver microsome incubations under ESI positive ionisation mode.

Metabolite description	ΔMass (Daltons)	[MH$^+$]	D/ND	t_R (min)	Metabolite Code
Parent	-	502	D	3.83	E209
Mono-oxygenation	+16	518	D	2.43	M+16 (I)
				2.88	M+16 (II)
				3.24	M+16 (III) (M only)
				3.96	M+16 (IV)
Deamination (alcohol)	-129	373	ND	-	-
Deamination of piperidine (alcohol)	-85	417	ND	-	-
Deamination of piperidine (acid)	-71	431	ND	-	-
Piperidine dealkylation	-86	416	ND	-	-
Piperidine ring opening (alcohol)	+18	520	ND	-	-
Ring dehydrogenation or Oxidative defluorination	-2	500	D	3.67	M-2
Tetroxane cleavage (ketone)	-182	320	D	0.99	M-182
Tetroxane cleavage (alcohol)	-180	322	D	1.01	M-180
Bis-oxygenation or Piperidine ring opening (acid)	+32	534	D	1.62	M+32 (I)
				2.91	M+32 (II) (H & R only)
				3.27	M+32 (III)
Ring dehydrogenations (x2)	-4	498	ND	-	-
Ring dehydrogenations (x3)	-6	496	ND	-	-
Mono-oxygenation & dehydrogenation	+14	516	ND	-	-

D: detected; ND: not detected

Supplementary Table 19: Metabolite profiles for E209 observed in human, rat and mouse liver microsomes incubations supplemented with NADPH.

Species	% Substrate Consumed	Relative Peak Area (expressed as % total metabolite peak area generated over 60 minutes)									
		M+16 (I)	M+16 (II)	M+16 (III)	M+16 (IV)*	M+32 (I)	M+32 (II)	M+32 (III)	M-182*	M-180	M-2
**Human**	45	**41**	**21**	ND	4	11	Trace	2	11	10	Trace
**Rat**	54	4	**20**	ND	**50**	2	Trace	3	13	9	2
**Mouse**	44	**27**	13	3	9	1	ND	Trace	**27**	**20**	Trace

ND: not detected; Trace: <1% total metabolite peak area

* M+16 (IV) and M-182 were also detected in control (non-cofactor) human, rat and mouse incubations suggesting a contribution of non-NADPH dependent metabolic pathways to the formation of these metabolites

Refer to Figure S8 for proposed metabolite structures

Supplementary Table 20: Cytochrome P450 Inhibition Potential of E209

Compound (Batch #)	IC_{50} (µM)					
	**CYP1A2**	**CYP2C9**	**CYP2C19**	**CYP2D6**	**CYP3A4** (Midazolam 1'-hydroxylation)	**CYP3A4** (Testosterone 6β-hydroxylation)
**Positive control inhibitors**	1.7 (furafylline)	0.71 (sulfaphenazole)	1.8 (ticlopidine)	0.023 (quinidine)	0.025 (ketoconazole)	0.035 (ketoconazole)
**E209**	>20 (n.m.i.)	>20 (n.m.i.)	>20 (n.m.i.)	>20 (n.m.i.)	>20 (n.m.i.)	>20 (n.m.i.)

n.m.i. No measurable inhibition of CYP activity observed at the highest concentration of the test compound used in the assay.

Supplementary Table 21: Estimated *in vitro* degradation half-lives in rat blood at 37°C.

Compound	Estimated degradation half-life (h)
**4**	14
**5**	15
**6**	12
**7**	15
**8**	16
**E209**	13
**JC-3-39**	4

Supplementary Note 1, Medicinal Chemistry Optimisation

Our initial strategy was to reduce the molecular weight of RKA182 to simplify the metabolic profile and improve PK properties. JC-3-39 performed well with improved PK profiles in the rat but assessment of the blood stability demonstrated comparatively poor overall stability. To increase lipophilicity, a series of *N*-functionalised analogues of JC-3-39 were prepared and assessed in *in vitro* metabolic and red blood cell stability assays, but none of these (MMV series 1) met the required stability profile set.

Supplementary Figure 4 describes the lead optimisation of MMV series 2. Insertion of a methylene spacer into **4** provided **5**; this modification increased *in vitro* potency and also saw an improvement in mean survival times to 16 days. However, *in vitro* metabolic stability and rat PK studies revealed a similar profile to **4** (Supplementary Table 1). The sulfonyl piperazine side chain had been explored in the OZ lead optimisation campaign[1] providing analogues with 30 day survival properties, thus analogues **6** and **7** were prepared (Supplementary Figure 4). The ethylene linked analogue **6** performed extremely well following a single oral dose of 30 mg/kg in the *P. berghei* model with an average of 19 day survival, had improved *in vitro* antimalarial potency, lower clearance values in rat liver microsomes, and improved PK performance in the rat (Supplementary Table 1). However, its intrinsic clearance in human liver microsomes was almost double that of analogues **4** and **5**. Sulfonamide **7**, the methylene extended analogue of **6**, had similar properties and the observed poor human microsomal stability suggests that the sulfonyl piperazine may be metabolically prone to specific human P450 isoforms. Difluroropiperidine analogue **8** and mono-fluoro piperidine E209 were designed as molecules with higher cLog P. E209 emerged as the best performing analogue in terms of *in vivo* potency with an MSD of 26.3 days with 2/3 animals cured after a single oral dose of 30 mg/kg (Table 1). The intrinsic clearance in human liver microsomes was also significantly improved compared with other analogues.

For the purpose of rat blood stability experiments, it was assumed that partitioning into erythrocytes was rapid and that slow partitioning did not contribute to the degradation profiles as indicated by the analysis of the plasma fraction. The half-life values shown in Supplementary Table 21 should be considered as estimates only, given the short sampling period relative to the estimated half-life values. The data indicate that the tetraoxane analogues depicted in Supplementary Figure 4 all had similar stability properties, with estimated half-lives of 13-16 h for each, while JC-3-39, the only compound lacking the 8'-phenyl group, showed a much shorter half-life of approximately 4 h.

In parallel, we also developed a series of benzylamine derivatives from which emerged N205 (**9**) (Supplementary Figure 4), a molecule that achieved a 66% cure rate following single dose treatment (1 x 30 mg/kg) in the *P. berghei* model and that performed slightly better than E209 in the *P. falciparum* SCID model, however, the overall exposure, metabolic stability and solubility characteristics (Supplementary Tables 1, 2 and 3) were inferior to E209 and this compound was not progressed further.

Supplementary Note 2 Synthesis of E209

The scale-up synthesis of E209 (Supplementary Figure 1) began with acetyl protection of the commercially available 4-(4-hydroxyphenyl)cyclohexanone **I** followed by formation of the gem dihydroperoxide **II**, under acid-catalysed conditions. This intermediate was then allowed to react with adamantan-2-one to provide a protected tetraoxane which was hydrolysed to phenol **III**. Alkylation of **III** with allyl bromide followed by ozonolysis provided aldehyde **IV**. Reductive amination of **IV** with 4-fluoropiperidine hydrochloride provided E209 as the free base.

Preparation of 4-(4-oxocyclohexyl)phenyl acetate II To a stirred solution of 4-(4-hydroxyphenyl)cyclohexanone (100.0 g, 526.3 mmol) and triethylamine (220 mL, 1.05 mol) in dichloromethane (1.0 L) was added acetic anhydride (107.0 g, 1.58 mol) dropwise) at 0 °C. The reaction mixture was then allowed to warm up to room temperature and stirred for 2h at rt. The reaction mixture was then washed with water (3 x 300 mL), saturated $NaHCO_3$ (2 x 300 mL) and brine (300 ml). The organic layer was dried with $MgSO_4$, filtered and concentrated under reduced pressure to give 4-(4-oxocyclohexyl)phenyl acetate (120.0 g, Yield: 98.3%) as a white solid. ^{1}H NMR (400 MHz, $CDCl_3$-d_6) δ_H 7.25 (d, 2H, *J* = 8.60 Hz, Ar), 7.04 (d, 2H, *J* = 8.60 Hz, Ar), 3.04 (tt, *J* = 3.34, 12.12 Hz, 1H, CH), 2.45-2.59 (m, 4H, CH_2), 2.30 (s, 3H, CH_3), 2.27- 2.15 (m, 2H, CH_2), 2.00-1.80 (m, 2H, CH_2); ^{13}C NMR (100 MHz, $CDCl_3$-d_6) δ_c 210.9, 169.6, 149.1, 142.3, 127.6, 121.6, 42.2, 41.3, 34.0, 21.1; LC-MS: *m/z* $[M + H]^+$ = 233, purity 98.63% (UV214 nm).

Synthesis of 4-(dispiro[cyclohexane-1,3'-[1,2,4,5]tetroxane-6',2''-tricyclo [3.3.1.1^{3,7}]decan]-4-yl)phenyl acetate To a stirred solution of 4-(4-oxocyclohexyl)phenyl acetate (30.0 g, 129.3 mmol) in acetonitrile (150 mL) and HCO_2H (150 mL) was slowly added 30% H_2O_2 (130 mL) at 0 °C. The reaction mixture was stirred at room temperature for 1hr. The mixture was added water (400 mL), extracted with DCM (3 x 300 mL). The combined organic layers were washed with water (3 x 300 mL), aqueous $NaHCO_3$ solution (150 mL) and brine (300 mL). The mixture was dried over $MgSO_4$, filtered and the filtrate **II** (about 800 ml) was used for next step without further purification. To a solution of II (800 mL) was added 2-adamantanone (25.2 g, 168 mmol) and $Bi(OTf)_3$ (4.24 g, 6.47 mmol). The mixture stirred for 1 hr at rt. The reaction mixture was filtered through a plug of silica and concentrated. Purification by column chromatography on silica gel to give 4-(dispiro[cyclohexane-1,3'-[1,2,4,5]tetroxane-6',2''-tricyclo[3.3.1.13,7]decan]-4-yl)phenyl acetate (20.0 g, Yield 37.3% two-step) as a white solid. ^{1}H NMR (400 MHz, $CDCl_3$-d_6) δ_H 7.22 (d, 2H, *J* = 8.50 Hz, Ar), 7.00 (d, 2H, *J* = 8.50 Hz, Ar), 3.48-2.92 (m, 2H), 2.70-2.49 (m, 1H, CH), 2.29 (s, 3H, CH_3), 1.48-2.13 (m, 20H, CH/CH_2); ^{13}C NMR (100 MHz, $CDCl_3$-d_6) δ_c 169.6, 148.9, 143.4, 127.8, 121.4, 110.5, 107.4, 46.9, 43.1, 39.2, 36.9, 33.1, 27.0, 21.1; MS (ES+), $[M + H]^+$ (100) 437.2 HRMS calculated for 437.1940 $C_{24}H_{30}O_6$ Na, found 437.1954. LC-MS: *m/z* $[M + Na]^+$= 437, purity 98.04% (UV214 nm).

Preparation of 4-(Dispiro[cyclohexane-1,3'-[1,2,4,5]tetroxane-6',2''-tricyclo[3.3.1.13,7] decan]-4-yl)phenol III To a solution of 4-(dispiro[cyclohexane-1,3'-[1,2,4,5]tetroxane-6',2''-tricyclo[3.3.1.13,7] decan]-4-yl)phenyl acetate (60.0 g, 144.9 mmol) in THF (500 mL) and water (200 mL) was added $LiOH.H_2O$ (18.2 g, 435 mmol). The reaction mixture was then stirred at rt for 2.0 h and then neutralised with dilute HCl. After carefully evaporation of most of the THF under reduced pressure, the mixture was extracted with DCM (2 x 300 mL). The combined organic layers were dried over $MgSO_4$, filtered and concentrated under reduced pressure. Purified by column chromatography on silica gel to give 4-(Dispiro[cyclohexane-1,3'-[1,2,4,5] tetroxane-6',2''-tricyclo[3.3.1.13,7] decan]-4-yl)phenol (45.0 g, yield 83.5%). ^{1}H NMR (400 MHz, $CDCl_3$-d_6) δ_H 7.09 (d, 2H, *J* = 8.50 Hz, Ar), 6.76 (d, 2H, *J* = 8.50 Hz, Ar), 4.73 (bs, 1H, OH), 3.47-2.99 (m, 2H, CH_2), 2.55 (tt, IH, *J* = 3.54, 11.55 Hz, CH), 2.10-1.56 (m, 20H, CH/CH_2); ^{13}C NMR (100 MHz, $CDCl_3$-d_6) δ_c 153.8, 138.2, 127.9, 115.2, 110.5, 107.6, ; 42.7, 36.9, 33.1, 27.0. LC-MS: *m/z* $[M + Na]^+$ = 395, purity 98.72% (UV214 nm).

Preparation of (1''*R*,3''*R*,5''*R*,7''*R*)-4-[4-(prop-2-en-1-yloxy)phenyl]dispiro [cyclohexane-1,3'-[1,2,4,5]tetroxane-6',2''-tricyclo[3.3.1.1^{3,7}]decane] To a solution of 4-(Dispiro[cyclohexane-1,3'-[1,2,4,5]tetroxane-6',2''-tricyclo [3.3.1.13,7] decan]-4-yl)-phenol (20.0 g, 53.8 mmol) in acetone (500 mL) was added potassium carbonate (37.0 g, 108 mmol) and allyl bromide (13.0 g, 108 mmol). The reaction mixture was heated to reflux for 24 h. The resulting suspension was cooled to rt, filtered to remove the solid and concentrated. Purification by column chromatography on silica gel to give (1''*R*3''*R*,5''*R*,7''*R*)-4-[4-(prop-2-en-1-yloxy)phenyl]dispiro[cyclohexane-1,3'-[1,2,4,5]-tetroxane-6',2''-tricyclo[3.3.1.1^{3,7}]decane] (21.5 g, Yield 97%) as a white solid. ^{1}H NMR (400 MHz, $CDCl_3$-d_6) δ_H 7.13 (d, 2H, *J* = 8.7 Hz, Ar), 6.85 (d, 2H, *J* = 8.7 Hz, Ar), 6.05 (ddt, 1H, *J* = 17.2,

10.6, 5.3 Hz, CH_2), 5.34 (ddd, 2H, *J* = 13.9, 11.9, 1.5 Hz, CH_2), 4.51 (dt, 2H, *J* = 5.3, 1.5 Hz, OCH_2), 2.66 (ddd, 1H, *J* = 15.4, 7.8, 3.8 Hz, CH), 2.09-1.53(m, 22H, CH_2/CH) MS (ES+), $[M + Na]^+$ (100) 435.2 LC-MS: *m/z* $[M + Na]^+$= 435, purity 98.37% (UV214nm).

Preparation of **(1''*R*,3''*R*,5''*R*,7''*R*)-4-[4-(but-3-en-1-yloxy)phenyl]dispiro[cyclohexane-1,3'-[1,2,4,5]tetroxane-6',2''-tricyclo[3.3.1.1^{3,7}]decane]** Prepared according to the procedure above with 4-bromobut-1-ene (White foam, 74%) ^{1}H NMR (400 MHz, $CDCl_3$-d_6) δ_H 7.13 (d, 2H, *J* = 8.7 Hz, Ar), 6.83 (d, 2H, *J* = 8.7 Hz, Ar), 5.97-5.83 (m, 1H, CH), 5.23-5.05 (m, 2H, CH_2), 3.99 (d, 2H, *J* = 6.7 Hz, CH_2), 2.60-2.48 (m, 3H, CH_2/CH), 2.10-1.55 (m, 22H, CH_2/CH) ^{13}C NMR (100 MHz, $CDCl_3$-d_6) δ_c 157.7, 138.5, 135.0, 1`28.2, 117.4, 114.9, 110.9, 108.0, 67.6, 43.2, 37.4, 34.7, 34.1, 33.6, 32.4, 30.4, 27.5 MS (ES+), $[M + Na]^+$ (100) 449.2.

Preparation of {4-[(1''*R*,3''*R*,5''*R*,7''*R*)-dispiro[cyclohexane-1,3'-[1,2,4,5]tetroxane-6',2''tricyclo[3.3.1.1^{3,7}]decan]-4-yl]phenoxy}acetaldehyde IV Ozone was bubbled through a solution of (1''*R*,3''*R*,5''*R*,7''*R*)-4-[4-(but-3-en-1-yloxy)phenyl]dispiro[cyclohexane-1,3'-[1,2,4,5]tetroxane-6',2''-tricyclo[3.3.1.1^{3,7}]decane] (10.0 g, 24.3 mmol) in methanol (20 mL) and DCM (160 mL) at -78 ^{o}C until the solution became saturated with ozone and appeared blue. Nitrogen was then bubbled through the solution for 20 min to purge excess ozone. DMS (7.52 g, 122.0 mmol) was added drop wise to the stirring solution at -78 ^{o}C. The mixture was stirred at -78 ^{o}C for 1 h, then allowed to warm up to rt and stirred for 1h. The reaction mixture was concentrated in vacuo to give crude product. Purification by column chromatography on silica gel gave {4-[(1''*R*,3''*R*,5''*R*,7''*R*)-dispiro[cyclohexane-1,3'-[1,2,4,5]tetroxane-6',2''-tricyclo[3.3.1.1^{3,7}]decan]-4-yl]phenoxy}acetaldehyde **IV**. ((12.0 g, White foam, 86%) ^{1}H NMR (400 MHz, $CDCl_3$-d_6) δ_H 9.86 (s, 1H, CHO), 7.17 (d, 2H, *J* = 8.7 Hz, Ar), 6.83 (d, 2H, *J* = 8.7 Hz, Ar), 3.57 (s, 2H, OCH_2), 2.66-2.52 (m, 1H, CH), 2.04-1.57 (m, 22H, CH_2/CH) (100 MHz, $CDCl_3$-d_6) δ_c 200.1, 156.5, 139.9, 128.4, 115.1, 110.9, 107.9, 96.4, 73.1, 70.7, 60.8, 55.8, 51.3, 43.2, 37.4, 33.6, 30.1, 27.5 MS (ES+), $[M + Na + CH_3OH]^+$ (100) 469.2 HRMS calculated for 469.2202 $C_{25}H_{34}O_7Na$, found 469.2216. LC-MS: *m/z* [M + Na + Methanol]$^+$ = 469, purity 92.87% (UV214nm).

Preparation of **3-{4-[(1''*r*,3''*r*,5''*R*,7''*R*)-dispiro[cyclohexane-1,3'-[1,2,4,5]tetroxane-6',2''-tricyclo[3.3.1.1^{3,7}]decan]-4-yl]phenoxy}propanal** Prepared according to the ozonolysis procedure above with (1''*R*,3''*R*,5''*R*,7''*R*)-4-[4-(but-3-en-1-yloxy)phenyl]dispiro[cyclohexane-1,3'-[1,2,4,5]tetroxane-6',2''-tricyclo[3.3.1.1^{3,7}]decane] (White foam, 68%) ^{1}H NMR (400 MHz, $CDCl_3$-d_6) δ_H 9.86 (s, 1H, CHO), 7.15 (d, 2H, *J* = 8.7 Hz, Ar), 6.84 (d, 2H, *J* = 8.7 Hz, Ar), 4.29 (t, 2H, *J* = 6.1 Hz, CH_2), 2.89 (dt, 2H, *J* = 6.1, 1.6 Hz, CH_2), 2.61-2.52 (m, 1H, CH), 2.10-1.53 (m, 22H, CH_2/CH) ^{13}C NMR (100 MHz, $CDCl_3$-d_6) δ_c 200.7, 157.3, 139.1, 128.3, 115.6, 114.9, 110.9, 108.0, 62.1, 43.7, 43.2, 37.4, 33.6, 30.2, 27.5 MS (ES+), $[M + Na + CH_3OH]^+$ (100) 482.2.

Preparation of 1-(2-{4-[(1''*R*,3''*R*,5''*R*,7''*R*)-dispiro[cyclohexane-1,3'-[1,2,4,5]tetroxane-6',2''-tricyclo[3.3.1.1^{3,7}]decan]-4-yl]phenoxy}ethyl)-4-fluoropiperidine To a solution of {4-[(1''*R*,3''*R*,5''*R*,7''*R*)-dispiro[cyclohexane-1,3'-[1,2,4,5]tetroxane-6',2''-tricyclo[3.3.1.1^{3,7}]decan]-4-yl]phenoxy}acetaldehyde **IV** (12.0 g, 24.3 mmol) in DCM (200 mL) was added 4-fluoropiperidine hydrochloride (5.06 g, 36.4 mmol) and the mixture was allowed to stir at rt for 1 h followed by the addition of sodium triacetoxyborohydride (10.3 g, 48.5 mmol). The resulting mixture was stirred at room temperature for 16 h and the pH adjusted to 8 with saturated aq. $NaHCO_3$, The mixture was filtered through celite. The filtrate was extracted with DCM (2 x 150 mL). The combined organic extracts were washed with brine (2 x 100 mL), dried over $MgSO_4$, filtered and concentrated in vacuo. The residue was purified by flash chromatography on silica gel to give 1-(2-{4-[(1''*R*,3''*R*,5''*R*,7''*R*)-dispiro[cyclohexane-1,3'-[1,2,4,5]tetroxane-6',2''-tricyclo[3.3.1.1^{3,7}]decan]-4-yl]phenoxy}ethyl)-4-fluoropiperidine **E209** (8.7g, yield 71.6%, two step) as a white solid. softens 60-62 ^{o}C ^{1}H NMR (400 MHz, $CDCl_3$-d_6) δ_H 7.14 (d, 2H, *J* = 8.6 Hz, Ar), 6.83 (d, 2H, *J* = 8.6 Hz, Ar), 4.86-4.64 (m, 1H, CHF), 4.17 (t, 2H, *J* = 5.4 Hz, OCH_2), 2.96 (t, 2H, *J* = 5.4 Hz, CH_2N), 2.89-

2.70 (m, 4H, NCH_2), 2.16-1.51 (m, 26H, CH_2/CH) (100 MHz, $CDCl_3$-d_6) δ_c 157.2, 139.0, 128.2, 114.9, 110.9, 107.9, 66.3, 65.5, 57.3, 49.7, 43.2, 37.4, 34.8, 33.6, 30.9, 27.5, 22.2 MS (ES+), $[M + H]^+$ (100) 502.3 HRMS calculated for 502.2969 $C_{29}H_{41}NO_5F$, found 502.2970. LC-MS: *m/z* $[M + H]^+$ = 502, purity 98.00% (UV214nm).

Preparation of Mesylate salt of E209 To a solution of **E209** (53.6g, 107.0 mmol) in diethyl ether (800 mL) and ethyl acetate (100 mL) were added methane sulfonic acid (12.3 g, 128 mmol) in diethyl ether (100 mL) drop wise. The resultant mixture was stirred at rt for 3 h. The precipitate formed was collected, washed with diethyl ether and air dried to give Mesylate salt 9 (58.5 g, Yield 91.6%) as a white solid. Mpt 161.6-162.5 °C ^{1}H NMR (400 MHz, DMSO-d_6) δ_H 9.54 (br. s., 1H), 7.19 (d, *J* = 8.71 Hz, 2H), 6.85 (d, 2H, *J* = 8.7 Hz), 4.82-5.00 (m, 1H), 4.24 - 4.37 (m, 2H), 3.45-3.68 (m, 4H), 3.02-3.32 (m, 4H), 2.56-2.71 (m, 1H), 2.33 (s, 3H), 1.45 - 2.29 (m, 24H); ^{13}C NMR (101 MHz, DMSO-d_6) δ_C 155.9, 138.8, 127.7, 114.7, 109.7, 107.3, 62.0, 54.9, 47.3, 41.4, 36.2, 32.6, 27.4, 27.2, 26.4 MS (ES+), $[M + H]^+$ (100) 502.3 HRMS calculated for 502.2969 $C_{29}H_{41}NO_5F$, found 502.2958 HRMS Elemental Analysis ($C_{30}H_{44}FNO_8S$), found C: 59.99 %, H: 7.36 %, N: 2.21 % (requires C: 60.28 %, H: 7.42 %, N: 2.34 %). LC-MS: *m/z* $[M + H]^+$= 502, purity 100.00% (UV 214nm).

The synthesis of tetraoxane analogues **4-8** is described in full in O'Neill et al. [1]

Supplementary Methods

In vitro parasite reduction ratio (PRR assays).[3]

In vitro parasite reduction ratio (PRR) testing was conducted at GlaxoSmithKline (Tres Cantos, Madrid, Spain) as previously described (Sanz et al. 2012).[3] The assay used the limiting dilution technique to quantify the number of parasites that remained viable after drug treatment. *P. falciparum strain* 3D7A (obtained from MR4 (Malaria Research and Reference Reagent Resource Center. http://www.beiresources.org) was treated with drug concentration corresponding to 10 x IC50. Conditions of parasites exposed to treatment were identical to those used at GSK in the IC50 determination (2% hematocrit, 0.5% parasitemia). Parasites were treated for 120 hours.

Drug in culture medium was renewed daily over the entire treatment period. Parasite samples were collected from the treated culture every 24 hours (24, 48, 72, 96 and 120 hour time points); drug was washed out of the sample, and parasites were cultured drug-free in 96 well plates by adding fresh erythrocytes and culture medium. To quantify the number of viable parasites after treatment, 3-fold serial dilutions were used with the above mentioned samples after removing the drug. Four independent serial dilutions were performed with each sample to correct experimental variations. The number of viable parasites was determined after 21 and 28 days by counting the number of wells with growth using [3H]-hypoxanthine incorporation. The number of viable parasites was back-calculated by using the formula Xn-1 where n is the number of wells able to render growth and X the dilution factor (when n = 0, number of viable parasites is estimated as zero). The PRR, defined as the logarithm to base 10 of the number of parasites the drug can kill in one parasite life cycle and indicating the killing rate of the compound investigated, was calculated as the decrease in viable parasites over 48 hours. A lag phase was considered to occur for as long as drug treatment did not produce the maximal rate of killing, and this period of time was excluded for PRR calculation.

In vitro metabolic stability of E209 in human, rat, mouse and dog liver microsomes and metabolite identification studies

Human, Dog, Rat and Mouse Microsomal Stability

Experimental Methods

Incubation methods:

The metabolic stability assay was performed by incubating E209 (1 and 5 µM for human, rat and mouse and 1 µM for dog) with human, dog, rat and mouse liver microsomes (Xenotech, Cat # H2640, D1000, R1000, M1000, respectively) in duplicate (only single determination for dog microsomes) at 37°C and 0.4 mg/mL protein concentration. The metabolic reaction was initiated by the addition of a NADPH-regenerating system and quenched at various time points over the 60 minute incubation period by the addition of acetonitrile. Control samples (containing no NADPH) were included (and quenched at 2, 30 and 60 minutes) to monitor for potential degradation in the absence of cofactor. Samples were analysed by UPLC-MS (Waters/Micromass Xevo G2 QTOF) under positive electrospray ionisation and MS spectral data acquired in a mass range of 80 to 1200 Daltons.

Metabolite identification (human, rat, and mouse microsomes) was performed using the 5 µM incubation samples with confirmation by accurate mass and MS/MS fragmentation where possible.

Calculations:

Test compound concentration versus time data were fitted to an exponential decay function to determine the first-order rate constant for substrate depletion. Each substrate depletion rate constant was then used to calculate: [1] a degradation half-life, [2] an *in vitro* intrinsic clearance value (CL_{int}, *in vitro*); [3] a predicted *in vivo* hepatic intrinsic clearance value (CL_{int}); [4] a predicted *in vivo* blood clearance value (CL_{blood}); and [5] a predicted *in vivo* hepatic extraction ratio (E_H).

[1] $$t_{1/2} = \frac{\ln(2)}{k}$$

[2] $$CL_{int,\,in\,vitro} = \frac{k}{\text{microsomal protein content (0.4 mg protein/mL)}}$$

[3]* $$CL_{int} = CL_{int,\,in\,vitro} \times \frac{\text{liver mass (g)}}{\text{body weight (kg)}} \times \frac{\text{mg microsomal protein}}{\text{g liver mass}}$$

[4]* $$CL_{blood} = \frac{Q \times CL_{int}}{Q + CL_{int}}$$

[5]* $$E_H = \frac{CL_{blood}}{Q} = \frac{CL_{int}}{Q + CL_{int}}$$

* The following scaling parameters[a] were assumed in the above calculations:

Species	Liver mass (g liver/kg body weight)	Microsomal protein (mg/g liver mass)	Hepatic blood flow (Q) (mL/min/kg)
Human	25.7	32	20.7
Dog	32.9	58	30.9
Rat	36.6	47	67.6
Mouse	54.9	47	120

[a] Ring et al. (2011) *Journal of Pharmaceutical Sciences*, 100:4090-4110.

This analysis is based on the assumption that the substrate concentration is below the apparent K_m for substrate turnover, that there is no significant product inhibition nor is there mechanism based enzyme inactivation, and that NADPH-dependent oxidative metabolism predominates over other metabolic routes.

Results and Discussion

Rates of Degradation

E209 exhibited degradation in human, rat and mouse liver microsomes with predicted hepatic extraction ratios being highest in humans and lowest in mice (Supplementary Table 9). Rates of degradation were similar at 1 and 5 µM in human, rat and mouse liver microsomes.

Metabolite Identification

A full metabolite search was conducted for E209 according to the metabolic transformations listed in Supplemetary Table 10 and the following metabolites were detected:

Four putative mono-oxygenation metabolites at $[MH^+]$ 518 (M+16 (I) to (IV)) were detected. MS/MS spectra for M+16 (I), (II) and (III) indicate hydroxylation on the adamantane ring (Supplementary Figure 8). The fragmentation pattern for M+16 (IV) suggests *N*-oxidation on the piperidine ring (Supplementary Figure 8).

Three putative bis-oxygenation metabolites at $[MH^+]$ 534 (M+32 (I) to (III)) were detected. MS/MS spectra for M+32 (I) indicate bis-hydroxylation on the adamantane ring (Figure S7). MS/MS spectra for M+32 (II, detected in human and rat microsomes only) and (III) suggest combined adamantane hydroxylation and piperidine *N*-oxidation (Supplementary Figure 8).

Two cleavage metabolites (M-182 and M-180) were detected in all three species and their structures were confirmed by MS/MS spectra (Supplementary Figure 8).

One putative metabolite with a molecular ion 2 Daltons less than the parent ($[MH^+]$ 500, M-2) consistent with either piperidine or hexane ring dehydrogenation or oxidative defluorination was also detected; however its MS signal was too weak to enable structural confirmation.

M+16 (IV) and M-182 were also detected in control (without cofactor) incubations suggesting a contribution of non-NADPH dependent metabolic pathways to the formation of these metabolites.

Comparing the metabolite profile across the three species (Supplementary Figure 6), hydroxylation(s) at the adamantane ring to M+16 (I) and/or (II) represent important metabolic pathways in human, rat and mouse liver microsomes. In addition, N-oxidation to M+16 (IV) appears to be an important metabolic pathway in rat whilst cleavage products represent significant metabolic pathways in mouse.

Tetraoxane Rat Blood Stability Studies

The study compared the stability of five tetraoxanes (**4-8** and E209) and comparator compoundJC-3-39 in freshly collected rat blood.

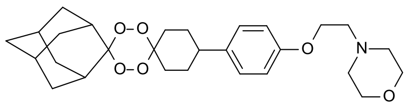	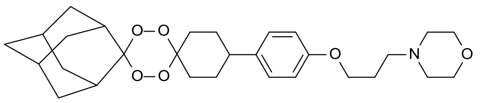
**NR-2-24 (4)**	**S201 (5)**
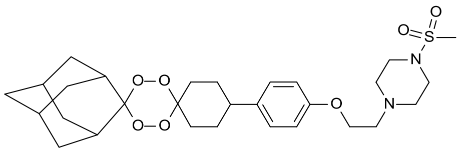	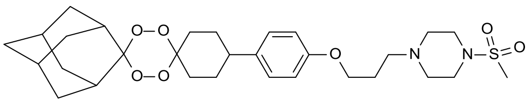
**S204 (6)**	**A217 (7)**
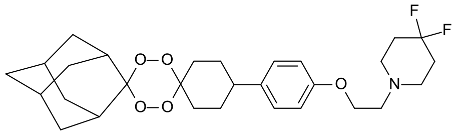	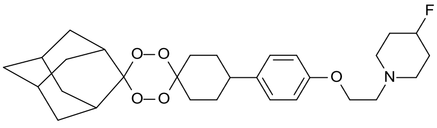
**E207 (8)**	**E209**
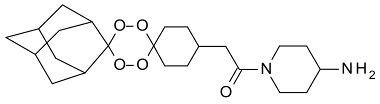	
**JC-3-39**	

Experimental Methods

For each compound, an aliquot of whole blood was collected from 7-9 week old male Sprague Dawley rats (pooled; n=2 rats) and spiked within 2 h of collection with a DMSO/acetonitrile/water solution to a nominal whole blood concentration of 1 µM. The final DMSO and acetonitrile concentrations were 0.2 and 0.4% (v/v), respectively. Aliquots of spiked whole blood were transferred to fresh microcentrifuge tubes and maintained at 37°C. At various time points over the 240 min, duplicate samples were centrifuged for 2 min and aliquots of the plasma fractions transferred to fresh microcentrifuge tubes and immediately snap frozen in dry ice. All plasma fraction samples were stored frozen (-20°C) until analysis.

The concentration of test compound in plasma samples was determined by UPLC-MS (Waters/Micromass Xevo triple quadrupole mass spectrometer) relative to calibration standards prepared in blank plasma. The lower limit of quantitation value for the plasma assay was 0.5 ng/mL for E209 and 1.0 ng/mL for the remaining seven compounds.

The apparent first order degradation rate constant (k) for each compound was estimated by linear regression of the log-transformed concentration-time data. The degradation half-life ($t_{1/2}$) was then calculated using the following equation:

$$t_{1/2} = \frac{\ln(2)}{k}$$

Results and Discussion

With the exception of JC-3-39, all other initial plasma concentrations were within approximately ±20% of the target spiked whole blood concentration. For JC-3-39, the initial plasma concentration was about 60% lower than the target spiked whole blood concentration which may be indicative of extensive partitioning into red blood cells.

The estimated degradation half-lives are listed in Supplementary Table 21. For the purpose of these experiments, it was assumed that partitioning into erythrocytes was rapid and that slow partitioning did not contribute to the degradation profiles as indicated by the analysis of the plasma fraction. The half-life values should be considered as estimates only given the short sampling period relative to the estimated half-life values.

The data indicate that the tetraoxane analogues all had similar stability properties, with estimated half-lives of 13-16 h for each. JC-3-39, the only compound lacking the 8'-phenyl group, showed a much shorter half-life of approximately 4 h.

Pharmacokinetic Analysis

Compartmental modelling was used to simultaneously fit oral and IV data generated in rats, mice and dogs and calculate bioavailability (expressed as F) as part of the process. Using a 3 compartment model as shown in the equations below, the plasma concentration versus time profiles were simulated. The differential equation system was solved using Pmetrics to predict the most likely parameters that fit the observed concentrations.

$$\frac{dgut}{dt} = -Gut \cdot ka \qquad \text{...Eq.S1}$$

$$\frac{d_{plasma}}{dt} = -\left[\frac{CL \cdot F}{V \cdot F} + \frac{Q_1 \cdot F}{V_{p1} \cdot F} + \frac{Q_2 \cdot F}{V_{p2} \cdot F}\right] \cdot Plasma + IV_{infusion} + ka \cdot Gut + \frac{Q_1 \cdot F}{V_c \cdot F} \cdot Peripheral_1 + \frac{Q_2 \cdot F}{V_c \cdot F} \cdot Peripheral_2 \qquad \text{... Eq.S2}$$

$$\frac{d_{Peripheral1}}{dt} = -\frac{Q_1 \cdot F}{V_c \cdot F} \cdot Peripheral_1 + \frac{Q_1 \cdot F}{V_{p1} \cdot F} \cdot Plasma \qquad \text{... Eq.S3}$$

$$\frac{d_{Peripheral2}}{dt} = -\frac{Q_2 \cdot F}{V_c \cdot F} \cdot Peripheral_1 + \frac{Q_2 \cdot F}{V_{p2} \cdot F} \cdot Plasma \qquad \text{... Eq.S4}$$

$$Plasma\ conc.(mg/L) = \frac{Plasma\ (mg)}{V_c\ (L)} \qquad \text{...Eq.S5}$$

Where ***Gut*** represents amount of drug in the gut (*mg*), $\boldsymbol{k_a}$ represents the rate constant for absorption from gut to plasma (hr^{-1}), ***Plasma*** represents amount of drug in plasma (*mg*), ***CL*** represents the clearance rate from the central plasma compartment (*L/hr*), V_c represents the central plasma volume of distribution (*L*), $\boldsymbol{Q_1}$ and $\boldsymbol{Q_2}$ respectively represent inter-compartmental clearance rates between plasma and the first and second peripheral compartments, respectively (*L/hr*), $\boldsymbol{V_{p1}}$ and $\boldsymbol{V_{p2}}$ represent the volumes of the first and second peripheral compartments (*L*), respectively, $\boldsymbol{IV_{infusion}}$ represents the infusion rate in plasma (*mg/hr*),

$\boldsymbol{F}$ represents the oral bioavailability compared to IV exposure, and $\boldsymbol{Plasma\ \ conc.}$ represents the drug concentration in plasma (mg/L).

Supplementary references

1 O'Neill, P.M. et al., Optimisation of the Synthesis of Second Generation 1,2,4,5 Tetraoxane Antimalarials, *Tetrahedron* **72**, 6118–6126, doi.org/10.1016/j.tet.2016.08.043 (2016)

2 Charman, S. A. *et al.* Synthetic ozonide drug candidate OZ439 offers new hope for a single-dose cure of uncomplicated malaria. *Proceedings of the National Academy of Sciences of the United States of America* 108, 4400-4405, doi:10.1073/pnas.1015762108 (2011).

3 Sanz, L. M. *et al.* P. falciparum in vitro killing rates allow to discriminate between different antimalarial mode-of-action. *PLoS One* **7**, e30949, doi:10.1371/journal.pone.0030949 (2012).

4. Chugh, M. et al. Identification and Deconvolution of Cross-Resistance Signals from Antimalarial Compounds Using Multidrug-Resistant *Plasmodium falciparum* Strains, Antimicrobial Agents and Chemotherapy *59*, 1110-1118, doi:10.1128/AAC.03265-14 (2015),
